# Mating-driven variability in olfactory local interneuron wiring

**DOI:** 10.1126/sciadv.abm7723

**Published:** 2022-02-18

**Authors:** Ya-Hui Chou, Chi-Jen Yang, Hao-Wei Huang, Nan-Fu Liou, Michael Raphael Panganiban, David Luginbuhl, Yijie Yin, Istvan Taisz, Liang Liang, Gregory S. X. E. Jefferis, Liqun Luo

**Affiliations:** 1 Institute of Cellular and Organismic Biology, Academia Sinica, Taipei 11529, Taiwan.; 2 Neuroscience Program of Academia Sinica, Academia Sinica, Taipei 11529, Taiwan.; 3 Genome and Systems Biology Degree Program, Academia Sinica and National Taiwan University, Taipei 10617, Taiwan.; 4 Howard Hughes Medical Institute and Department of Biology, Stanford University, Stanford, CA 94305, USA.; 5 Department of Zoology, University of Cambridge, Cambridge CB2 3EJ, UK.; 6 Neurobiology Division, MRC Laboratory of Molecular Biology, Cambridge CB2 0QH, UK.

## Abstract

Variations in neuronal connectivity occur widely in nervous systems from invertebrates to mammals. Yet, it is unclear how neuronal variability originates, to what extent and at what time scales it exists, and what functional consequences it might carry. To assess inter- and intraindividual neuronal variability, it would be ideal to analyze the same identified neuron across different brain hemispheres and individuals. Here, using genetic labeling and electron microscopy connectomics, we show that an identified inhibitory olfactory local interneuron, TC-LN, exhibits extraordinary variability in its glomerular innervation patterns. Moreover, TC-LN’s innervation of the VL2a glomerulus, which processes food signals and modulates mating behavior, is sexually dimorphic, is influenced by female’s courtship experience, and correlates with food intake in mated females. Mating also affects output connectivity of TC-LN to specific local interneurons. We propose that mating-associated variability of TC-LNs regulates how food odor is interpreted by an inhibitory network to modulate feeding.

## Teaser

An identified olfactory interneuron exhibits extraordinary sexually dimorphic variability in connectivity patterns.

## INTRODUCTION

The nervous system comprises numerous neuron types. A given neuron type can further exhibit variability in morphology, connectivity, neurotransmitters, and ion channel compositions, adding complexity to the brain connectome (*1*–*9*). Such neuronal variability may be the combined result of stochasticity in developmental processes (*2*, *3*, *10*) and experience-dependent functional connectivity (*11*, *12*) of the nervous system. The extent to which a single neuron can exhibit wiring variability remains largely unknown. The only way to directly address this question is to compare the connectivity of identified neurons, such as the large ocellar interneuron in grasshopper (*13*) or the Mauthner cell in zebrafish (*14*), across many individuals. This would provide the ultimate single-cell resolution for studying wiring variability and is the goal of this study.

## RESULTS

### TC-LN exhibits extraordinarily variable connection patterns

**Fig. 1.**
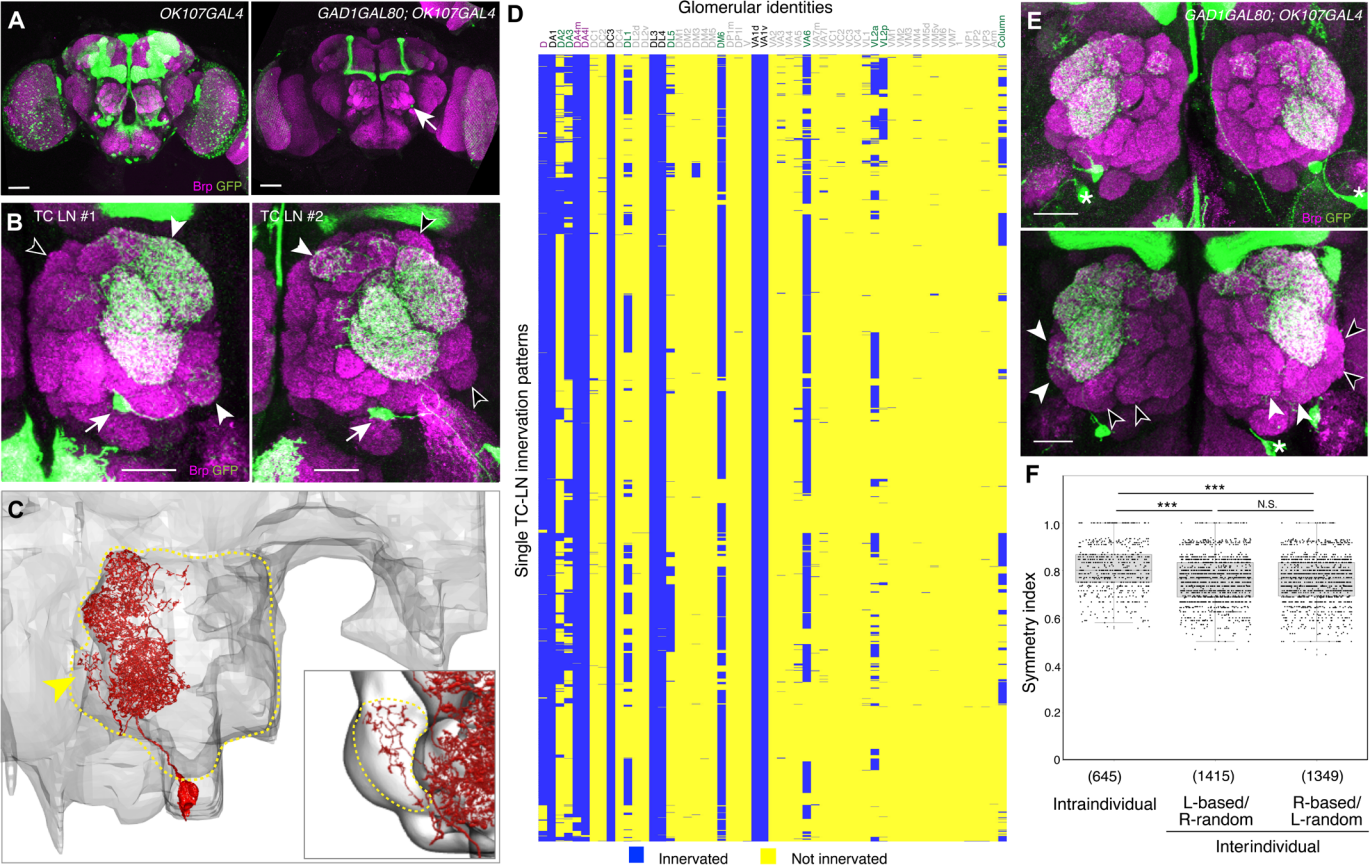
Variable glomerular innervation patterns of the TC-LN. (**A**) Adult brains of flies carrying *OK107-GAL4* (left) or *GAD1-GAL80;OK107-GAL4* (right) were stained with a neuropil marker, Bruchpilot (Brp; magenta), and mCD8GFP (green) expressed under *UAS* control. Only one olfactory local interneuron, TC-LN, was labeled in the right antennal lobe (arrow, right image). Scale bars, 50 μm. (**B**) Two high-magnification images of TC-LNs from two different brains showing variable innervation patterns. Filled and open arrowheads indicate glomeruli that are innervated and not innervated by the TC-LNs, respectively. Soma are indicated by arrows. Scale bars, 20 μm. (**C**) Only one TC-LN (vLN2-36_R; 5813054716) was identified in the intact antennal lobe of the hemibrain EM connectome. Arrowhead indicates processes in VL2a. Dashed line contours the antennal lobe. Inset shows the innervation in the VL2a glomerulus (dashed outline) from this TC-LN. (**D**) Hierarchical clustering of the innervation patterns derived from 2764 TC-LNs of flies with a single genetic background *y*^1^ w*. Glomeruli always innervated (black), with innervation frequency >95% (magenta) and between 10 and 84% (green), are color-coded. (**E**) TC-LNs in two hemispheres of the same fly may exhibit the same (top) or partially different (bottom) innervation patterns. Filled and open arrowheads indicate glomeruli innervated and not innervated, respectively. Asterisks indicate non-LN neurons that innervate neuropils other than antennal lobes. Scale bars, 20 μm. (**F**) Symmetry indices of TC-LNs in left and right antennal lobes of the same brains and randomly paired brains show intra- and interindividual variability. See Materials and Methods for details of the symmetry index. The numbers of pairs are shown in parentheses. Gray boxes indicate the 25 to 75th percentile range; whiskers indicate 75th percentile + 1.5 interquartile range and 25th percentile – 1.5 interquartile range. Wilcoxon rank sum test was used to assess paired data. ***P < 0.001; N.S., not significant.

We have previously shown that antennal lobe local interneurons (LNs) in the *Drosophila* olfactory system exhibit a high degree of wiring variability as a group (*5*). To consistently visualize the same single neuron, we developed a method combining a *GAL4* and a *GAL80* transgene, such that, in ~60% of the brain hemisphere, just one LN was labeled (Fig. 1A, fig. S1, and Materials and Methods). *Drosophila* olfactory receptor neurons (ORNs) expressing the same odorant receptor converge their axons to a single glomerulus in the antennal lobe, where they synapse with their cognate projection neurons (PNs) to relay olfactory information to higher brain centers (*15*). Of the 55 glomeruli in the antennal lobe, representing 55 discrete olfactory processing channels (*16*, *17*), this LN consistently innervated almost all glomeruli that are targets of trichoid ORNs (Fig. 1B); therefore, we named the neuron TC-LN. TC-LN could be unambiguously identified by its consistent and dense innervations of nine glomeruli (see below). We also examined two *Drosophila* electron microscopy connectome datasets (*18*, *19*), covering three brain hemispheres, and found just one TC-LN in each antennal lobe (Fig. 1C and fig. S2). Thus, our method allowed us to consistently label a single identified neuron per hemisphere.

We used the binary glomerular innervation patterns of LNs (*5*) as a simple metric to assess the wiring variability of TC-LN. By determining the innervation patterns of 3300 TC-LNs of flies with different age (ranging from 0-2 hours to 23 days old), sex, mating status, and genetic background, we found that TC-LNs exhibited an extremely high degree of wiring variability (Fig. 1, B and D, fig. S3, and table S1). Each TC-LN innervated 6 to 24 of 55 glomeruli (mean ± SD = 12.67 ± 1.89, *n* = 3300), with 609 different innervation patterns across individuals of the same genetic background (*n* = 2764; Fig. 1D) and 849 distinct patterns across all individuals examined (fig. S3A). However, the variability of TC-LN innervation patterns was not random. Six glomeruli were always innervated, and three additional glomeruli were innervated by more than 95% of TC-LNs, comprising a defining characteristic of TC-LNs. In contrast, nine additional glomeruli were variably innervated, with frequencies between 10 and 84% (fig. S4A, table S1, and Supplementary Note).

Having demonstrated that variability is nonrandom, we further examined the relationship between the innervation density and variability. We chose eight glomeruli—four always innervated (DA1, VA1d, VA1v, and DA4m), two mostly innervated (D and DM6), and two that were innervated in a smaller subset of the samples (VL2a and DL1). We found that the four glomeruli always innervated had the highest innervation density, followed by the two mostly innervated glomeruli, and VL2a and DL1 had the lowest innervation density (fig. S5A and table S2). For glomeruli with low innervation density, density-based quantification often assigned innervations to samples that did not have innervation by manual expert curation (fig. S5B), likely because of insufficient background subtraction (Materials and Methods). Therefore, we used manual binary scoring for the rest of the study.

The variability of TC-LNs was observed in newly eclosed flies (fig. S3C), suggesting that developmental stochasticity contributes to TC-LN innervation variability. Such stochasticity-caused variability persisted in adults (intraindividual variability; Fig. 1, E and F, intraindividual variability). Among 2764 brains with labeled TC-LNs in the *y*^1^ w* background, 645 brains had one labeled TC-LN in each antennal lobe, allowing us to compare TC-LNs between two brain hemispheres of the same animal (Fig. 1E). Hierarchical clustering revealed that the TC-LN pairs in the left and right hemispheres of the same brain also exhibited morphological variability (fig. S6, A and B). However, the intraindividual variability was lower than the interindividual variability (Fig. 1F and fig. S6C), suggesting that individual experience also contributes to TC-LN innervation variability.

### Sexual dimorphism contributes to TC-LN morphological variability

**Fig. 2.**
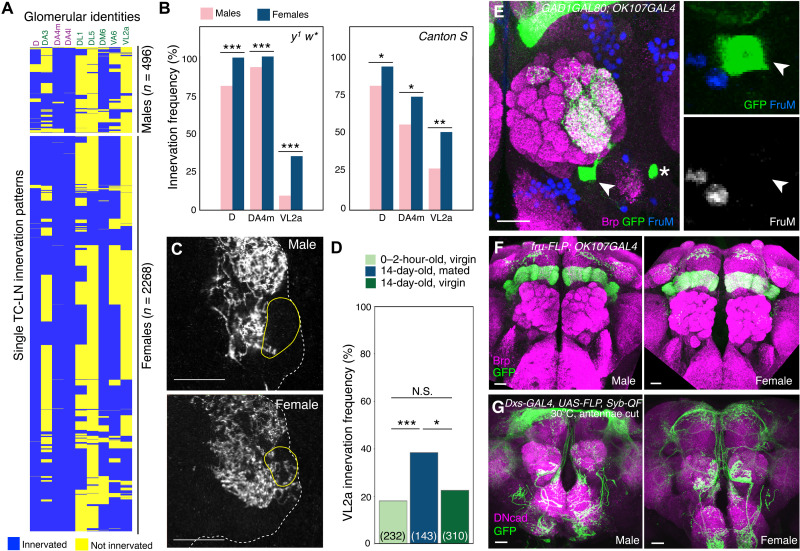
TC-LN innervation pattern exhibits sexual dimorphism and is modulated by mating. (**A**) Hierarchical clustering of 496 TC-LNs of male and 2268 TC-LNs of female flies with the genetic background *y*^1^ w* in nine glomeruli with significantly different innervation frequencies in both sexes. Glomeruli with 100 or 0% innervation in both sexes were excluded. Chi-square tests, followed by post hoc Bonferroni correction, were conducted to examine the differences between males and females in 43 glomeruli (table S3). *P < 0.05; **P < 0.01; ***P < 0.001; N.S., not significant. (**B**) Innervation frequencies of three sexual dimorphic glomeruli of TC-LNs of flies with the genetic background *y*^1^ w* (left; *n* = 496 from males and 2268 from females) and *Canton S* (right; *n* = 123 from males and 214 from females). (**C**) Partial projection images of antennal lobes (dashed lines) that cover glomerulus VL2a (solid lines) in male (top) and female (bottom) brains. Scale bars, 20 μm. (**D**) Sexually dimorphic VL2a innervation of TC-LNs was enhanced by female courtship experience. Parentheses show the total number of cells examined. Chi-square test, followed by Bonferroni correction, was conducted to examine the differences between virgins and mated females in 26 glomeruli in *y*^1^ w* (table S5). *P < 0.05; **P < 0.01; ***P < 0.001. N.S., not significant. (**E** and **F**) TC-LNs do not express Fru^M^. (E) A male brain with TC-LN (green) was stained for Fru^M^ (blue). (F) *fru-FLP* was used to capture possible transient expression of Fru^M^ during development. Both male and female brains lacked Fru^M^ expression in developmental history (see table S9 for full genotypes). Scale bars, 20 μm. (**G**) Intersection expression driven by *dsx-GAL4* was used to capture possible transient expression of *dsx* during development. Both male and female brains lacked developmental *dsx* expression (see table S9 for full genotypes). Scale bars, 20 μm.

Because TC-LNs consistently innervate target glomeruli of trichoid ORNs, which mainly detect pheromones (*20*, *21*), we next compared the innervation profiles of TC-LNs from females and males (Fig. 2A, table S3, and Materials and Methods). Of the 43 glomeruli that received innervation in a subset of TC-LNs that we sampled, three glomeruli—D, DA4m, and VL2a—exhibited sexually dimorphic innervation frequencies in both *y*^1^ w* and *Canton S* strains (Fig. 2B; fig. S4, B to D; and tables S3 and S4). We focused on VL2a in our subsequent study because it is one of three sexually dimorphic glomeruli that are innervated by ORNs and PNs that express a male-specific form of *fruitless*, FruM (*22*). TC-LNs innervated VL2a more often in females (34.9%) than in males (9.3%) (Fig. 2, B and C). While VL2a ORNs sense food odors, VL2a PNs project axons to the pheromone-specific regions of the lateral horn (*23*, *24*).

*fruitless* (*fru*) and *doublesex* (*dsx*) are two master regulators of sexually dimorphic circuits in *Drosophila* (*22*, *25*–*27*). Because VL2a is a sexually dimorphic glomerulus innervated by Fru^M^-positive ORNs and PNs, we tested whether the sexual dimorphism of TC-LNs is regulated by Fru or Dsx. Published profiles of all Fru-positive or Dsx-positive sexually dimorphic neurons in adult brains do not include TC-LNs (*28*–*30*). Therefore, we asked whether TC-LNs might transiently express Fru and/or Dsx during development. We found that neither protein was expressed in TC-LNs (Fig. 2, E to G, and fig. S7), suggesting that Fru and Dsx do not cell-autonomously regulate TC-LN sexual dimorphism. TC-LNs in virgin and mated females innervated similar sets of glomeruli (Supplementary Note). However, in mated females, TC-LNs had a specific and significantly higher VL2a innervation frequency than virgins (Fig. 2D and table S5, top); this was not observed in D and DA4m (fig. S4E and table S5, top). These properties were specific to females and were not observed in males (fig. S4F and table S5, bottom). These results suggested that female mating experience modifies the TC-LN innervation pattern.

### Variable VL2a innervations of TC-LNs correlate with feeding events of mated females

To investigate the mechanisms of TC-LN morphological changes in mated females, we assessed VL2a innervation under different conditions. Because TC-LNs from mated females more frequently innervate VL2a, we first sought to determine whether male-derived substances during mating might drive female TC-LN innervation in VL2a. During mating, males transfer to females (i) sperm; (ii) sex peptide (SP), which modulates female’s post-mating behaviors (*31*); (iii) *ci*s-vaccenyl acetate (cVA), a lipid produced by male to inhibit mating from other males (*32*); and (iv) male-derived cuticular hydrocarbons that are sent to females through contact (*33*). We used males lacking SP (*31*), sperm [*bol* mutants (*34*)], or cuticular hydrocarbons [oenocyte-less mutants (*35*)] to mate with control females and analyzed the innervation patterns of TC-LNs of those females (fig. S8 and table S6). We found that sperm, SPs, and cuticular hydrocarbons were all individually dispensable for the increased frequency of VL2a innervation by TC-LNs. These findings suggest that increased VL2a innervation in mated females is likely driven by a combination of multiple factors during mating.

**Fig. 3.**
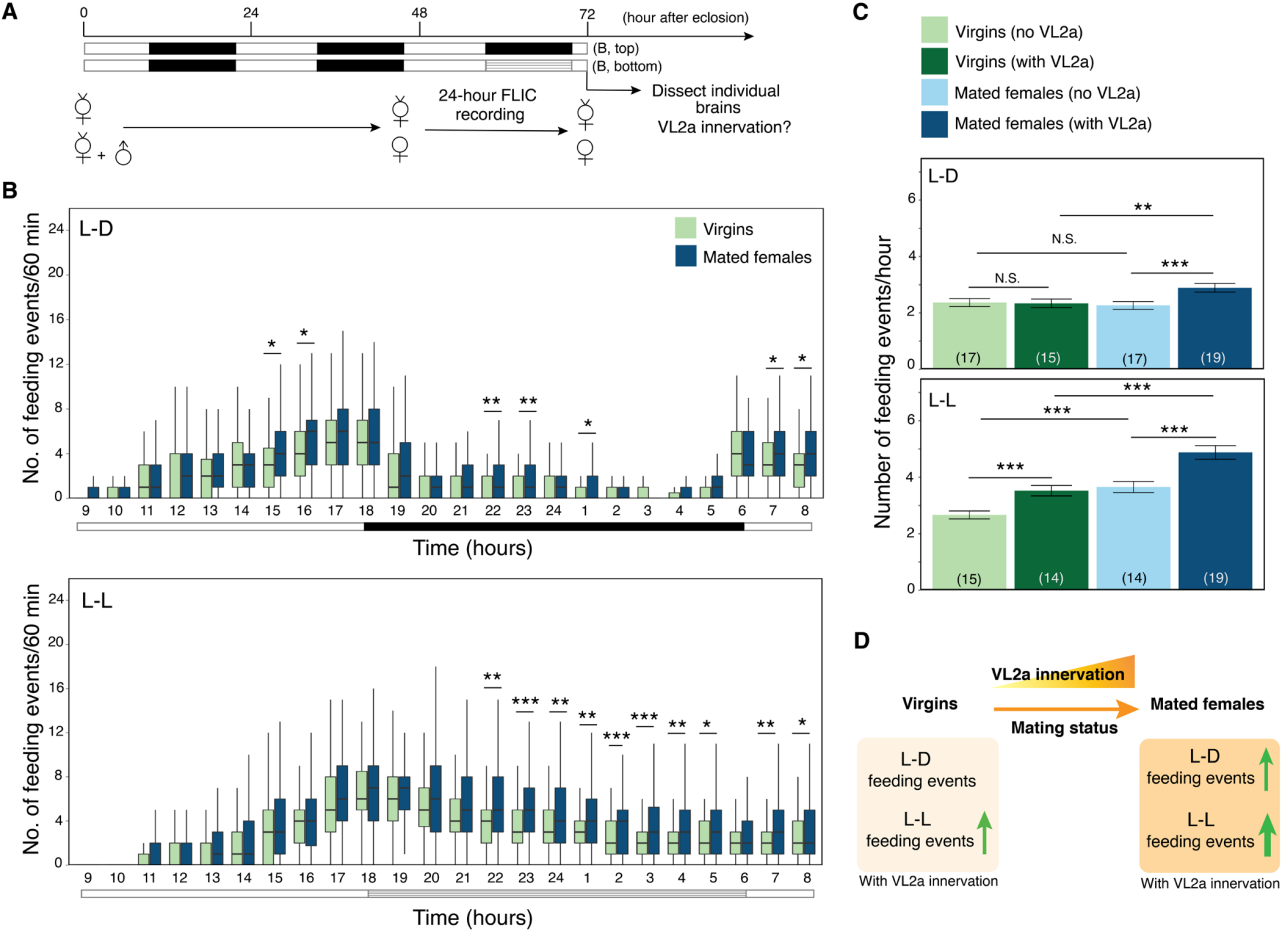
VL2a innervation by TC-LNs correlates with rhythmic eating behavior of mated females. (**A**) Experimental diagram of FLIC analysis. (**B**) Feeding events (within 24 hours) of virgins and mated females under light-dark (L-D) or constant light (L-L) conditions. Hatched bar marks the subjective night in the L-L group. Two-way ANOVA was used to examine the main effects and interactions of variables, followed by post hoc Bonferroni correction of multiple pairwise comparisons among different conditions (tables S7 and S8). Statistical significance was determined after correction as *P < 0.05; **P < 0.01; ***P < 0.001. (**C**) The relationship between mating status, VL2a innervation by TC-LNs, and feeding events under L-D and L-L conditions. Three-way ANOVA was used to examine the main effects and interactions of variables (table S7). *P < 0.05; **P < 0.01; ***P < 0.001; N.S., not significant. (**D**) Summary of TC-LN innervation in VL2a glomerulus. Mating enhances the frequency of female TC-LN innervation in VL2a. This change correlates with increase of feeding events in mated females. When overall feeding events are increased under the L-L condition, VL2a innervations also correlate with the increased feeding behaviors in virgins and mated females.

After mating, females exhibit behavioral changes including mating receptivity, egg laying, food preference, and feeding behavior (*36*). Mated females consume more food than virgins to produce eggs (*37*). In addition, VL2a is innervated by food-sensing ORNs that are thought to coordinate feeding with reproductive behaviors (*24*). We therefore asked whether VL2a innervations might be related to changes in feeding behavior of mated females using the fly liquid-food interaction counter (FLIC) system (fig. S9 and Materials and Methods) (*38*). Newly eclosed virgin females were divided into two groups, in the presence or absence of sibling virgin males, for 2 days (Fig. 3A). Individual virgins or mated females were subsequently subjected to the FLIC test and then examined for TC-LN innervation patterns. Consistent with previous reports (*36*, *37*), mated females ate more than virgins (Fig. 3B, top). Furthermore, mated females with TC-LN innervation in VL2a tended to eat more than virgins with VL2a innervation; this difference was not observed in mated females and virgins without TC-LN innervation of VL2a (Fig. 3C, top; fig. S10, left panels; and tables S2, S7, and S8).

Feeding is timed by circadian rhythms, which are known to regulate synaptic connection strengths (*39*). Because flies ate more during the light phase in the standard light-dark (L-D) rearing condition described above (Fig. 3B, top), we reasoned that shifting them to constant light (L-L) might enhance their feeding. This experimentally induced feeding enhancement may allow us to test the causality between TC-LN VL2a innervation and female’s eating behavior. We observed three effects on feeding under the L-L condition [Fig. 3, B (bottom) and C (bottom); fig. S10, right panels; and tables S2, S7, and S8]. First, both virgins and mated females enhanced their eating under L-L compared to L-D. Second, mated females still ate more than virgins. Third, flies with VL2a innervation ate more in both groups.

The L-L females experienced the same light-dark condition as L-D females in the first 2 days and 9 hours until the ninth hour under FLIC test (Fig. 3A). If VL2a innervation in virgins changed due to higher eating frequencies right before brain dissection, we would expect to see higher VL2a innervation frequencies in L-L virgins than in L-D virgins. Yet, the VL2a innervation frequencies were comparable for L-L (41.2%, *n* = 34) and L-D (46.9%, *n* = 32) virgins. On the other hand, if VL2a innervation occurred before the FLIC test and, in turn, enhanced feeding behavior in L-L virgins, we should observe a positive correlation between VL2a innervation and eating behavior, which was the case. Similar feeding enhancement by TC-LN VL2a innervation was also observed in L-L mated females, given comparable VL2a innervation frequencies under L-L (56.1%, *n* = 41) and L-D (56.4%, *n* = 39). These results suggested that VL2a innervation enhances feeding rather than the other way around. Under the L-L condition, VL2a innervation by TC-LN might act on top of mating status and enhance the feeding of virgins (Fig. 3D).

### Changes of TC-LN local circuit connection in mated females

**Fig. 4.**
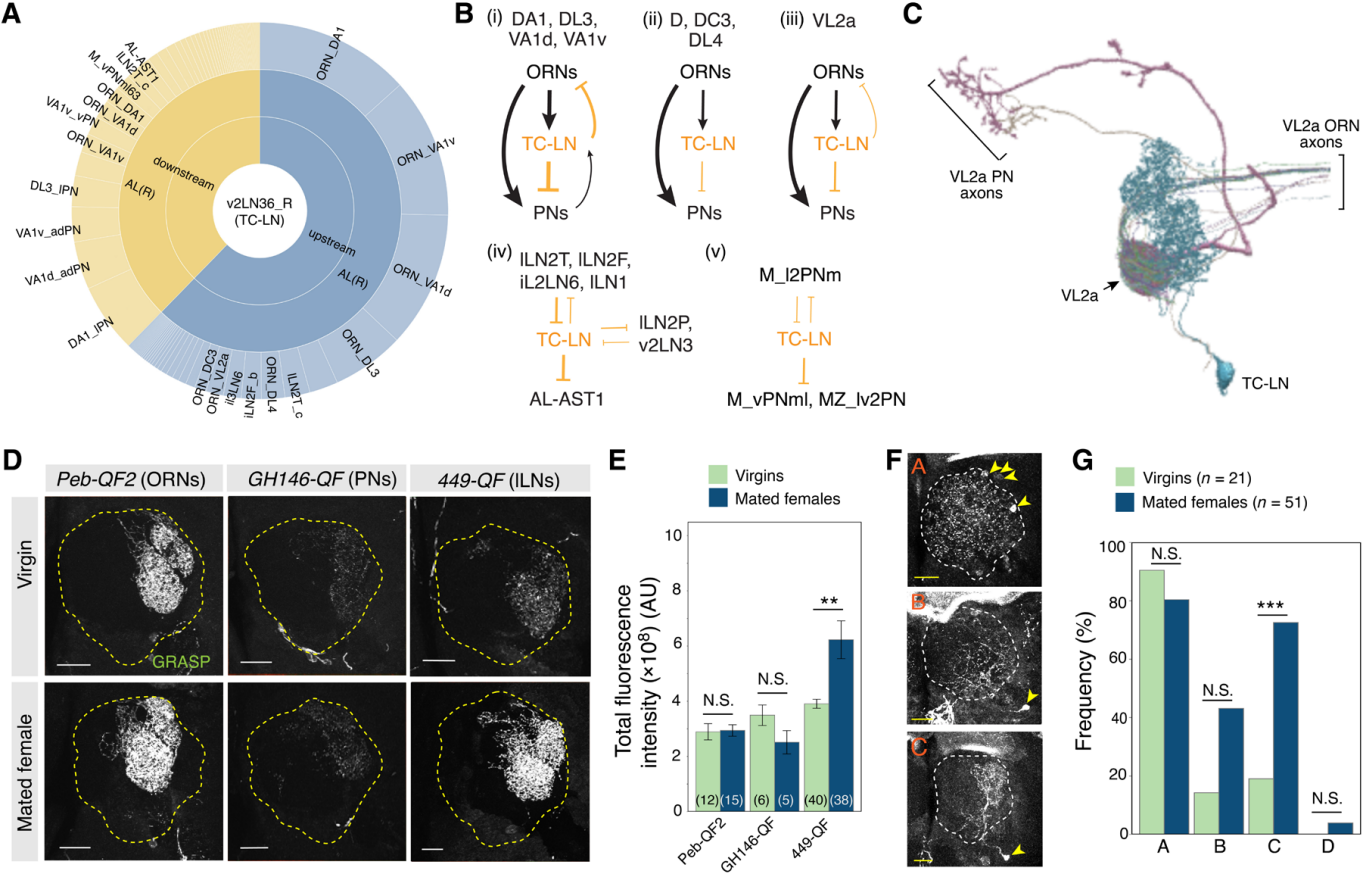
Mating experience changes female TC-LN synaptic connectivity. (**A**) Input and output neurons of TC-LN (v2LN36_R neuron, 5813054716) identified from the hemibrain v1.2 dataset (adapted and modified from neuprint.janelia.org). (**B**) Summary of the TC-LN connectome. The thickness of arrows and lines approximates the input and output number between indicated neurons and TC-LN in a given glomerulus (i to iii) or between indicated types of neurons and TC-LN (iv and v). Black arrows and orange lines indicate excitation and inhibition signals, respectively. Although a small subset of LNs is excitatory, for simplicity, LNs are generally assumed to be inhibitory. (**C**) VL2a connectome of TC-LN, showing traces of all VL2a ORNs (gray) and two of four PNs targeting VL2a (a uni-glomerular PN in magenta and a multi-glomerular PN in gold) in addition to TC-LN (cyan). (**D**) GRASP signal between ORNs and TC-LN (left), between PNs and TC-LN (middle), or between lateral unilateral LNs and TC-LN (right) in brains of 7-day-old females. (**E**) Quantification of fluorescence intensity representing GRASP signal in the antennal lobe. Parentheses are numbers of antennal lobes with TC-LN examined. Two-way ANOVA with a post hoc Bonferroni correction for multiple comparison was used. (**F**) Three major types of postsynaptic neurons of TC-LNs are uncovered by *trans*-Tango in brains of 8-day-old females. More than one type of neuron may be uncovered in an antennal lobe. See fig. S13E for details of the experimental scheme. (**G**) Quantification of TC-LN postsynaptic neurons in virgin and mated females. Cell types A, B, and C are based on those in (F). Type D are cells that did not fall into type A, B, or C. *n* indicates the number of TC-LNs examined. Chi-square test, followed by Bonferroni correction, was used to examine the differences between pairs. Scale bars, 20 μm. **P < 0.01; ***P < 0.001; N.S., not significant.

To better understand the relationship between variable innervation of VL2a by TC-LNs and post-mating feeding behaviors of females, we next examined TC-LN’s synaptic connection patterns. Immunostaining revealed that TC-LN is glutamatergic (fig. S11, A and B). It has previously been shown for some LNs of the ventral v2 group to which TC-LN belongs that glutamate has an inhibitory action through glutamate-gated chloride channels (*40*). Thus, TC-LN likely contributes to local inhibition in the antennal lobe. Like most unilateral LNs, TC-LN has presynapses evenly distributed along all processes (fig. S11C). The electron microscopy (EM)–reconstructed TC-LN (v2LN36_R, cell ID: 5813054716) has 7315 input connections, which are ORN rich (heavily biased to ipsilateral ORNs), and 6310 output connections, which are PN rich (Fig. 4A and fig. S11, D and E) (*18*). The connectivity patterns vary in different glomeruli for this TC-LN. Focusing on single-glomerular ORNs and PNs, in glomeruli DA1, VA1d, VA1v, and DL3, this TC-LN forms reciprocal connections with both ORNs and PNs (Fig. 4B). In glomeruli DL4, DC3, and D, TC-LN mostly receives input from ORNs and sends output to PNs. Last, in glomerulus VL2a, TC-LN receives input mostly from ORNs but sends minimal output to PNs or back to ORNs (Fig. 4, B and C, and figs. S11, D and E, and S12). Such differences may reflect different amounts of TC-LN arbors and synapses in these glomeruli (Fig. 4A and figs. S5A and S11F). This TC-LN also receives strong input from multiple LNs (Fig. 4, A and B, and fig. S11G) and sends output to different LN populations and to multi-glomerular PNs (Fig. 4, A and B, and fig. S11H).

While EM connectomics provides a great amount of detail, it cannot be used for studying structural plasticity due to the small numbers of specimens at present. To test for possible changes in connectivity between TC-LN and ORNs, PNs, or LNs in mated females, we performed green fluorescent protein (GFP) reconstitution across synaptic partners (GRASP) (fig. S13A) (*41*). We found labeled ORNs, PNs, and LNs (Fig. 4D), consistent with the EM reconstruction data (Fig. 4, A to C). Contacts between TC-LN and ~60 LNs (*42*) from the lateral lineage, but not between TC-LN and ORNs or PNs, were enhanced in mated females (Fig. 4, D and E; fig. S13, B to D; tables S2 and S6; and Supplementary Note). Because GRASP does not distinguish pre- and postsynaptic contacts, we next applied *trans*-Tango (*43*) to reveal changes specifically in TC-LN postsynaptic partners (fig. S13E). More than 51% TC-LN synaptic outputs were to ORNs, PNs, and multi-glomerular PNs. To have a better resolution of possible changes in LNs, we used a stringent condition of *trans*-Tango, in which only postsynaptic neurons with stronger connections to TC-LNs would be visualized (see Supplementary Note). We found that TC-LNs of mated females tended to have stronger connections to some postsynaptic neurons, especially one type of ventral LNs (Fig. 4, F and G; figs. S11I and S13, F and G; and table S6). These results collectively suggested that the TC-LN local circuit, especially its connections to other LNs, was changed in female brains after mating.

## DISCUSSION

We found that an identified olfactory LN exhibits as many as 849 distinct glomerular innervation patterns in different brain hemispheres, demonstrating an extraordinary level of variability in its synaptic connections. Because each glomerulus receives inputs from a distinct ORN type representing a specific set of olfactory cues, the distinct innervation patterns of TC-LNs imply differential integration of olfactory information. Specifically, it has been previously shown that IR84a ORNs sense food odor and relay information to glomerulus VL2a, which integrates food and mating behaviors in males (*24*). On the basis of our data, we propose that TC-LN within the dynamic VL2a local connectome in female brains serves as a central switch to tune odor-sensing toward specific food odor and modulate eating behaviors of mated females (see also Supplementary Note for possible recruited TC-LN synaptic neurons in mated females).

The malleability of TC-LN wiring further suggests that LNs may be more flexible and dynamic than hard-wired sensory and projection neurons (*17*, *44*), consistent with previous observations of bulk LN plasticity induced by strong odor stimulations (*45*–*47*). It will be interesting to explore in the future whether distinct interneurons with different circuit functions exhibit different degrees of neural variability and whether such variability can be a substrate for evolutionary differences in olfactory function across species.

## MATERIALS AND METHODS

### Flies

Flies were reared on standard cornmeal-agar-glucose-sucrose medium and maintained at 25°C in an incubator with 12 hour–12 hour L-D cycle and about 50% relative humidity. Flies used in this study were *y*^1^ w*, *Canton S* (Bloomington *Drosophila* Stock Center, BDSC 1), *Oregon-R* (isogenic, gift from T. Clandinin), *UAS-nuclacZ* (*48*), *UAS-mCD8GFP.1* (*48*), *UAS-FRT-stop-FRT-mCD8GFP* (*49*), *UAS-CD4GFP1-10* (*50*), *QUAS-CD4::GFP11* (this study), *UAS-syt-HA (III)*, *QUAS-mtdTomato-3xHA* (*49*), *13xlexAOP2-mCD8GFP (attp40)* (BDSC 32205) (*51*), *GAD1-GAL80 (#11-2)* (this study), *tub-GAL80[ts]* (*52*), *tub-Arrestin-TEV* (*53*), *fru-FLP* (gift from B. Dickson) (*30*), *hsFLP12*, *OK107-GAL4* (BDSC 854), *OK371-GAL4* (BDSC 26160), *fru-GAL4* (gift from B. Dickson) (*22*), *DvGlut[CNSIII]-GAL4* (gift from A. DiAntonio) (*54*), *GH146-QF* (*49*), *peb-QF2* (BDSC 66474) (*55*), *449-QF* (*56*), *P[trans-Tango]attP40* (BDSC 77124) (*43*), *SPR-Tango-VG (#6)* (gift from S. Kondo) (*57*), *dsx-GFP.FPTB* (BDSC 51966) (*58*), *w*^1118^ (BDSC 5905), *fru[2]* (gift from D. Yamamoto) (*59*), *fru[sat]* (gift from D. Yamamoto) (*59*), *SP[CTL]* (gift from B. Dickson) (*31*), *SP[0325]* (gift from B. Dickson) (*31*), *bol[1]* (BDSC 11794), *SPR[2A]* (BDSC 84576) (*60*), *Mip[2A]* (gift from Y. Rao) (*60*), and oenocyte-less mutants (gift from J. Levine) (*35*). Full genotypes of flies used in this study are listed in table S9.

### Flies for TC-LN variability

Unless otherwise indicated, flies used for visualizing TC-LNs were in *y*^1^ w* single genetic background and with the same genotype as “*y*^1^ w*;GAD1-GAL80 (#11-2)/*UAS-nuclacZ UAS-mCD8GFP;;OK107-GAL4/+*” or in *Canton S* single genetic background (BDSC 1) and with the same genotype as “*w^1118^;GAD1-GAL80 (#11-2)*/*UAS-mCD8GFP.1;;OK107-GAL4/+*” (tables S1 and S9). Virgin siblings from the same bottle were used for control and experimental groups, so rearing conditions were the same. In general, around 14 to 25 flies (either all virgins, all virgin males, or virgins and virgin males) were group-housed in a food vial with a diameter of 2.5 cm. In some pilot tests, five virgins, five virgin males, or five virgins and three virgin males were group-housed in a 2.5-cm food vial. No obvious differences in TC-LN innervation variability were observed between these two culture conditions. To create a single genetic background, individual transgenes or mutant alleles were backcrossed to the particular genetic background at least five times. When collecting TC-LNs from flies of different ages, the flies were flipped to a fresh vial every 2 to 3 days.

### *GAD1-GAL80* transgenic flies

A 3.098-kb fragment containing the upstream regulatory region of *GAD1* gene was amplified from the genomic DNA of *GAD1-GAL4* flies (*61*) by polymerase chain reaction (PCR) with the following primers: *GAL45pR* (TCGGTTTTTCTTTGGAGCAC) and *White3pF* (TTCCGCAAAAATGGGTTTTA). This fragment was cloned into *pCR4-TOPO* using a TOPO TA cloning kit (Invitrogen, K457502) to generate *pCR-GAD1p*. The *GAL80* coding sequence was isolated from *pCasper4-tubulin-GAL80* (*48*) and cloned into the backbone of *pCasperAug-GAL4-X* (gift from L. Vosshall) using Not I and Xba I sites to build *pCaSper4-GAL80* containing *AdhAUG*. Last, the *GAD1* regulatory fragment was cut from *pCR-GAD1p* and cloned upstream to *AdhAUG-GAL80* in *pCaSpeR4-GAL80* using Not I sites to generate *pCaSpeR4-GAD1-GAL80*. The construct was microinjected into *w*^1118^ embryos to create *GAD1-GAL80* transgenic flies. Four independent lines were established. Through combinations with *NP3056-GAL4*, *OK107-GAL4*, and *GH146-GAL4*, it was determined that all four *GAD1-GAL80* lines showed extensive ectopic expression of GAL80 in γ-aminobutyric acid (GABA)–negative neurons of adult brains. The intersection experiments using *GAD1-GAL80 (#11-2)* and *OK107-GAL4* consistently produced labeling of one particular LN in the antennal lobe, with rare exceptions; the labeled neuron is called TC-LN in this study (see fig. S1).

### *QUAS-CD4-spGFP11* and *QUAS-CD4-spGFP1-10* transgenic flies

The *CD4-spGFP11* and *CD4-spGFP1-10* fragments were amplified from genomic DNA of *LexAop-CD4::spGFP11* and *UAS-CD4::spGFP1-10* flies (*50*), respectively, through polymerase chain reaction (PCR) with the primer pair, *spGFP-F* (CCCTCGAGATGCCACCTTCAACATCATTG) and *spGFP-R* (GCTCTAGACTAGCGCCTTCGGTGCCG). The fragments were cloned to *pCR4-TOPO* through a TOPO TA cloning kit (Invitrogen, K457502) to generate *pCR-CD4-spGFP11* and *pCR-CD4-spGFP1-10*, respectively. The *CD4-spGFP11* and *CD4-spGFP1-10* fragments were cut from *pCR-CD4-spGFP11* and *pCR-CD4-spGFP1-10* and respectively cloned into *pQUAST* (*49*) using Xba I *and* Xho I sites to build *pQUAS-CD4-spGFP11* and *pQUAS-CD4-spGFP1-10*. The constructs were microinjected into *w*^1118^ embryos to create the corresponding *QUAS-CD4-spGFP11 and QUAS-CD4-spGFP1-10* transgenic flies.

### Glomerular innervation profiling

The original confocal stacks of brains from three to five different experimental groups were first scrambled and coded by one researcher (Stephanie Wheaton, D.L., Ying-Jun Chen, or H.-W.H.). Y.-H.C. blindly scored all TC-LN innervation profiles. The cell identities of scored results were then decoded. The innervation profiles of single TC-LNs were blindly scored on the basis of 55 glomeruli in the antennal lobe. A glomerulus was scored as 1 when one or more process longer than ~3 μm innervated the glomerulus, or 0 when it was not innervated. The binary TC-LN innervation profiles were subjected to hierarchical clustering through MultiExperiment Viewer (MeV, 4.8.1) (http://mev.tm4.org/#/welcome) (*62*), with optimized gene leaf order, Pearson correlation, and complete linkage clustering.

### Immunohistochemistry

Immunostaining was performed as previously described (*42*). Adult brains were dissected in 4% paraformaldehyde (Electron Microscopy Sciences, 15713-S) in phosphate-buffered saline (PBS) and fixed in the same fixative at room temperature (RT) for 20 min with rotation. Brains were washed three times with PBST (0.3% Triton X-100 in 1× PBS) at RT for 20 min each wash, followed by blocking in 5% normal goat serum (NGS; Jackson ImmunoResearch Laboratories, 005-000-121) in PBST at RT for 30 min. Samples were incubated with primary antibodies (Abs) in 5% NGS/PBST at 4°C for two to three overnights. After three 20-min PBST washes at RT, brains were incubated with secondary Abs in PBST at 4°C for one to two overnights, followed by three 20-min PBST washes at RT. Samples were soaked in SlowFade Gold Antifade Mountant (Invitrogen, S36936) for at least one overnight at 4°C before mounting on the slide. Primary Abs used were as follows: mouse anti-Bruchpilot (Brp) [1:25, Developmental Studies Hybridoma Bank (DSHB), nc82], mouse anti-V5 (1:150; Invitrogen, 11417489), mouse anti-GFP (3E6, 1:500 for GRASP; Invitrogen, A-11120), rat anti-mCD8 (1:100; Invitrogen, MCD0800), rat anti-HA (3F10, 1:200; Roche, 11867423001), rat anti–DN-cadherin (1:20; DSHB, DN-Ex#8), rabbit anti-FruM^21^ (1:3000; gift of B. Dickson), rabbit anti-GABA (1:200; Sigma-Aldrich, A2052), rabbit anti-DvGLUT (*54*) (1:1000; a gift from A. DiAntonio), rabbit anti-HA (1:500 to 1:1000; ab9110, Abcam), rabbit anti-GFP (1:1000; Invitrogen, A11222), and rabbit anti-GFP (1:1000; Invitrogen, A6455). Secondary Abs used were goat anti-mouse IgG, goat anti-rat IgG, and goat anti-rabbit IgG, which are conjugated with Alexa Fluor 488, DyLight 488, Cy3, Cy5, Alexa Fluor 647, or DyLight 649. All secondary Abs were from Jackson ImmunoResearch Laboratories or Invitrogen and used at 1:500. Brains were imaged using a Zeiss LSM510 confocal microscope, a Zeiss LSM780 confocal microscope equipped with Mai Tai HP-1040 (Spectra-Physics), or a Zeiss LSM700 confocal microscope and processed using AimImageExaminer (Carl Zeiss), ZEN black (Carl Zeiss), Fiji (https://fiji.sc), and Adobe Photoshop (Adobe).

### Antennal lobe segmentation and TC-LN neurite density analyses

To quantify the neurite density of TC-LN in individual glomeruli, single glomeruli were manually segmented in Fiji (ImageJ) and then processed in Python and R. Briefly, 15 antennal lobes were randomly selected through a custom R script from each of four groups [virgin females, mated females, virgin males, and mated males in the *y*^1^ w* background (Fig. 1D and table S1)], with total 60 antennal lobes. From the randomly selected antennal lobes, one expert manually defined the boundary of each glomerulus using the Bruchpilot channel on single z slices, blind to GFP signal, at an interval of one to three slices using a custom workflow in Fiji (https://imagej.net/software/fiji/). The boundaries in the remaining z slices were then obtained by interpolation to generate a three-dimensional binary mask with the same shape as the original confocal stack. The binary masks were then subjected to our Python script to calculate the total intensity of the GFP channel of voxels within the mask boundaries. In addition to VA1d, VA1v, DA1, DA4m, DM6, D, DL1, and VL2a (fig. S5), DM5, a glomerulus that is never innervated by TC-LN, was also segmented to serve as an internal reference to background in the GFP channel for each antennal lobe.

After obtaining the total GFP channel intensity in segmented glomeruli, the subsequent calculations were performed in R. To eliminate background levels in the GFP channel, the average background value per voxel for each antennal lobe was acquired using the ratio of total GFP channel fluorescence intensity in DM5 divided by the total voxel count of DM5. To obtain the background-adjusted total signal, the total signal obtained for each glomerulus was background-adjusted by subtracting the product of background value per voxel and the total voxel count of the given glomerulus (Eq. 1.1). To eliminate other batch effects within each antennal lobe, an internal normalizing constant was defined as the total background-adjusted fluorescence signals of VA1d, VA1v, and DA1 glomeruli, which are consistently densely innervated by TC-LN (fig. S5A). For each glomerulus, the normalized total fluorescence signal was obtained by dividing the background-adjusted total signal by the internal normalizing constant for its respective antennal lobe. The normalized fluorescence signal of each glomerulus was then divided by the voxel count of the glomerulus to obtain the process density (Eq. 1.2)

$${\left(\text{total signal}\right)}_{x}^{\prime }={\left(\text{total signal}\right)}_{x}-\frac{{\left(\text{total signal}\right)}_{\text{DM}5}}{{\left(\text{voxel count}\right)}_{\text{DM}5}}{\left(\text{voxel count}\right)}_{x}$$ (1.1)

$${\left(\text{neurite density}\right)}_{x}=\frac{{\left(\text{total signal}\right)}_{x}^{\prime }/\sum_{i=\{\text{VA}1d,\text{VA}1v,\text{DA}1\}}{\left(\text{total signal}\right)}_{i}^{\prime }}{{\left(\text{voxel count}\right)}_{x}}$$ (1.2)

### Identifying sexual dimorphic innervation of TC-LNs

To identify glomeruli that exhibit sexual dimorphic innervations from TC-LN, the innervation frequencies of individual glomeruli by TC-LN from females (*n* = 2268) and males (*n* = 496) in the *y*^1^ w* background were subjected to chi-square test, followed by adjusting *P* values through post hoc Bonferroni correction for multiple comparison using the “p.adjust” R package. Only glomeruli with nonzero and nonunity innervation frequencies were subjected to chi-square test and post hoc Bonferroni correction. This resulted in 43 glomeruli of flies in the *y*^1^ w* background (fig. S4D and table S3). Similar analyses were used to identify the sexual dimorphic innervation of TC-LNs of flies in the *Canton S* background (*n* = 214 and 123 TC-LNs from females and males, respectively). Twenty-eight glomeruli with nonzero and nonunity innervation frequencies were subjected to the analyses (fig. S4D and table S3). It is recognized that the chi-square is highly sensitive in calling statistical differences in the *y*^1^ w* group due to its large sample size. Nonetheless, three glomeruli—D, DA4m, and VL2a—exhibit significant differences between two sexes in both *y*^1^ w* and *Canton S*.

### GFP reconstitution across synaptic partners

Newly eclosed virgins and virgin males were collected and reared either as virgin groups of the same sex in a vial or as mated groups, in which virgins and virgin males were reared together in a vial. The flies were aged at 25°C, ~50% relative humidity in a 12 hour–12 hour L-D–controlled incubator for 7 days. Adult brains were dissected and stained with Abs as described above. All images were collected under the same confocal configuration. The raw confocal stacks only covering the antennal lobes were processed with Fiji (https://imagej.net/software/fiji/) to measure the sum of fluorescence intensity and to project the confocal stack into a single image. Antennal lobes were contoured on the basis of the Brp signal. The total fluorescence intensity of a given antennal lobe was calculated by multiplying the mean fluorescence intensity per pixel by the total area (in pixels) of that antennal lobe.

### *trans*-Tango

Newly eclosed virgins and virgin males were collected and reared either as virgin groups of the same sex in a vial or as mated groups, in which virgins and virgin males were reared together in a vial. The flies were aged at 25°C, ~50% relative humidity in a 12 hour–12 hour L-D–controlled incubator for either 7 or 8 days. Adult brains were dissected and stained with Abs as described above. All images were collected under the same confocal configuration. No obvious differences in TC-LN expressions and *trans*-Tango signals were observed between brains from 7- and 8-day-old flies in the same experimental groups and across trials. Therefore, samples from 7- and 8-day-old flies were grouped together for statistical analysis.

### Intersectional studies of Fru-positive neurons

To identify any interneurons that may express *fru-GAL4*, 0- to 5-day-old flies were dissected and immunostained to visualize *fru-GAL4*–positive neurons (antennae intact group; fig. S7B). In the second group, both antennae of 0- to 1-day-old adult flies were removed with forceps (Dumont No. 5 biology, 11252-20), followed by a 7-day recovery at 25°C to allow for the elimination of degenerated antennal ORN axons (antennae cut group; fig. S7B). For the intersectional experiments using *Fru-FLP* and *OK107-GAL4*, *OK371-GAL4*, or *DvGLUT-GAL4*, the flies were raised at 25°C throughout all developmental stages. Under this condition, only *fru*-positive neurons would express FLP, which binds to the two FRT sites of the flip-out reporter and removes the stop codon, thus producing a functional *UAS-FRT-mCD8GFP* expression construct. The GAL4 expressed by corresponding *GAL4* drivers then binds to *UAS* and drives *mCD8GFP* expression (Fig. 2F and fig. S7C). Full genotypes can be found in table S9.

### Intersectional studies of Dsx-positive neurons

The flies were raised at 18°C throughout all developmental stages (18°C, antennae intact group; fig. S7F) or at 18°C for 6 days (from embryonic to approximately second instar larval stage) and then transferred to 30°C until adult stage (30°C, antennae intact group; fig. S7F). Adult brains of 0- to 3-day-old males and females were dissected and immunostained to identify neurons that had expressed *Dsx-GAL4* at any time during development or adulthood. To eliminate ORN axons and allow visualization of possible antennal lobe innervations of other neurons, 0- to 3-day-old males and females from the 30°C group were collected, and both antennae were removed with forceps, followed by an 8-day recovery at 25°C to allow for the elimination of degenerated antennal ORN axons (30°C, antennae cut group; Fig. 2G and fig. S7F). Full genotypes can be found in table S9.

### Oenocyte-less flies

Cuticular sex pheromones are long-chain hydrocarbons produced by oenocytes. Control and oenocyte-less flies were established as described (*35*). Briefly, 0- to 1-day-old flies were collected and group-housed at 25°C for 1 day. The vials with flies were transferred to 30°C overnight and then back to 25°C for about 12 hours. The vials were then similarly cycled through 30° and 25°C an additional three times. The flies were then flipped to a new vial and kept at 25°C for 2 to 3 days. Right before establishing the cross, control and oenocyte-less males were anesthetized on ice for ~10 min and quickly examined under fluorescence microscope to verify the ablation of oenocytes in putative oenocyte-less flies. Flies were allowed to recover at 25°C for several hours to 1 day before crossing with newly eclosed virgins carrying TC-LNs.

### *bol* mutant males

*bol^1^* homozygous males lack any detectable Boule expression in testis; thus, *bol^1^* is a strong or null allele of *boule* (*34*). The fertility of *bol^1^/+* and *bol^1^* males was examined. After each cross, eggs laid on the food vial were cultured at 25°C for 5 days to allow hatching of progenies. All vials with *bol^1^/+* males contained hatched larvae. A large portion of vials with *bol^1^* homozygous mutant males did not contain any hatched larvae, but around one-third of vials did. Accordingly, females crossed with *bol^1^* mutant males were separated as *bol^1^*-fertilized and *bol^1^*-sterile groups (fig. S8C).

### FLIC analysis

The *Drosophila* feeding monitors (DFMs) and master control unit (MCU) of the FLIC modules were purchased from Sable Systems International and assembled with food reservoirs and a high-speed charge-coupled device camera (GZL-CL-22C5M-C, Point Grey), with a 1″ 8mm SWIR C-Mount lens (LM8HC-SW, Kowa American Corp.), in the laboratory (fig. S9A). The liquid food, 10% sucrose (w/v; S1888, Sigma-Aldrich) solution, was freshly prepared and loaded into four 50-ml syringes connected to two DFMs (fig. S9A). All experiments were conducted in a designated behavior room, which was constantly controlled at 25°C, 55% relative humidity and absolute darkness. A set of white lights was present above the FLIC system to control L-D conditions. In brief, female flies were briefly ice-anesthetized and placed individually into single FLIC arenas, followed by a 20-min recovery period. Detected FLIC signals were analyzed with a FLIC algorithm in R (Sable Systems International) to identify feeding events.

To establish the threshold of true feeding events, videos (30 frames per second) and FLIC signals (200 ms per signal) derived from single flies were synchronized and coupled through a custom-written script in MATLAB (MathWorks). L-D reared 1- to 7-day-old mated *Canton-S* females were subjected to 6-hour FLIC test 5.5 hours after light on and under L-L conditions. Only signals derived from contact of the proboscis and the food reservoir were counted as true feeding. The rate of false-positive signals was 2.17%, derived mostly from occasional touching of legs or wings. On the basis of the analysis of 46 events from three flies, the threshold of true feeding signals was set as 25 arbitrary units (AU). Accordingly, a true feeding event was defined as five consecutive data points with background signal–subtracted values of ≥25 AU (fig. S9, B and C).

To determine the single genetic background of flies carrying TC-LNs for FLIC analysis, 1- to 7-day-old *Canton S* and *Oregon R* mated females were subjected to the FLIC test. Experiments were started between 12:15 and 18:40 under 12 hour–12 hour L-D conditions. On the basis of the total feeding events, average intensity, and maximal intensity of 12 *Canton S* (total of 413 feeding events) and 12 *Oregon R* flies (total of 375 feeding events) (fig. S9D), *Canton S* was selected for the genetic background for TC-LNs.

To collect TC-LN innervation patterns from single flies after the FLIC test, newly eclosed virgins and virgin males were collected by researcher #1 (Y.-H.C.). Virgins were divided into two equal groups and then group-housed as virgins only (10 to 14 virgins) or group-housed with virgin males (10 to 14 virgins and 20 virgin males) in a food vial and under 12 hour–12 hour L-D for 2 days. Subsequently, mated and virgin female flies were briefly ice-anesthetized and placed individually into FLIC chambers by researcher #2 (C.-J.Y.). Following a 20-min recovery in the chamber, the FLIC signals were collected for 24 hours beginning at 3 hours after lights on. During the FLIC test, flies were kept under either 12 hour–12 hour L-D or constant light L-L condition. At the conclusion of the FLIC test, the entire DFM was transferred to 4°C and incubated for 20 min to anesthetize the fly. Individual virgins and mated female flies were transferred to single 1.5-ml tubes, coded, and scrambled by researcher #2. Coded flies were then passed to researcher #1, who was blind to the treatment conditions, for individual dissection and immunostaining of the adult brain. Confocal images of individual brains were collected and glomerular innervations of TC-LNs were scored by researcher #1. The scoring results were then decoded by researcher #2 to assign the mating status of individuals.

### Correlation analysis

Pearson correlation (*r*) was used to evaluate the relationship between two glomeruli (fig. S4, B and C)

$$r=\frac{\Sigma_{i=1}^{n}(x_{i}-\bar{x})(y_{i}-\bar{y})}{\sqrt{\Sigma_{i=1}^{n}{\left(x_{i}-\bar{x}\right)}^{2}\Sigma_{i=1}^{n}{\left(y_{i}-\bar{y}\right)}^{2}}}$$

where *x_i_* and *y_i_* are the *i*th number of two sequences, and $\bar{x}$ and $\bar{y}$ are the respective means of the two sequences. In our case, *x_i_* and *y_i_* were either 0 or 1. If the glomerulus *x* was innervated by TC-LN in the *i*th brain, *x_i_* is 1. Otherwise, *x_i_* is 0. Correlation was used to measure the similarity of two glomeruli, in terms of how frequently the two glomeruli were innervated by the same TC-LN. For example, a correlation close to 1 means that the two glomeruli are very frequently innervated by the same TC-LN. A correlation close to −1 means that one glomerulus tends not to be innervated when the other glomerulus is innervated by the TC-LN. A correlation close to 0 means that there is no obvious relationship between the two glomeruli.

### Asymmetry index and random pairing

The assignment of left and right antennal lobes was from the observer’s view. Therefore, the cells labeled as “right” are in the left brain hemispheres of the fly, and vice versa. The symmetry index was designed to quantify the similarity of innervation patterns between the TC-LNs in two brain hemispheres of the same brain or between two randomly paired TC-LNs. The definition of the symmetry index is as follows: Two sets of glomeruli were considered; one was collected from a brain hemisphere innervated by a TC-LN, and the other from a second brain hemisphere innervated by a second TC-LN. The two brain hemispheres were taken as a pair. Let *x* be the number of glomeruli representing the intersection of these two sets, and let *y* be the number of glomeruli in the union of the two sets. Accordingly, the symmetry index is *x*/*y*.

Random pairing of one right TC-LNs with one left TC-LN from any other brain was performed as follows. The number of left brain hemispheres is *n*, and the number of right brain hemispheres is *m*, where *m* is less than *n* and *n* is less than 2 *m*. TC-LNs from *m* left brain hemispheres were randomly chosen to pair with TC-LNs from *m* right brain hemispheres. Pairs of two TC-LNs from the same brain were excluded. This procedure yielded R-based/L-random interindividual pairs (Fig. 1F and fig. S6C). Similarly, *n*-*m* TC-LNs were additionally chosen from the original *m* right brain hemispheres and combined to the *m* right brain hemispheres to make *n* right brain hemispheres. These *m* right brain hemispheres were then randomly chosen to pair with the *n* left brain hemispheres. Pairs of two TC-LNs from the same brain were excluded. This procedure generated *n* pairs of L-based/R-random interindividual pairs.

To analyze possible differences of asymmetry indices between left and right TC-LNs in the same brains (intraindividual) or the left and right TC-LNs from different brains (interindividual), asymmetry indices derived from three groups (Fig. 1F) were subjected to the Kruskal-Wallis test, followed by Wilcoxon rank sum test.

### Statistical analysis of TC-LN innervations after FLIC

Feeding events from different trials were pooled and binned into 1-hour time bins. Statistical analyses were performed using R. Two-way analysis of variance (ANOVA) and post hoc Bonferroni correction were used to examine the differences between all virgins and all mated females in L-D or L-L (Fig. 3B, fig. S10A, and tables S7 and S8). Briefly, two-way ANOVA was first used to examine whether the two variables, mating status and time bin, had main effects on the feeding events of virgins and mated females. Only the test results that showed main effects were subjected to post hoc analysis for pairwise comparison with Bonferroni correction. The statistical significance of differences was determined through this correction. To further test the contribution of VL2a on flies’ feeding events, FLIC data derived from flies carrying labeled TC-LNs were used to examine the interactions and main effects of three variables: mating status, time bin, and VL2a innervation. In short, these data were subjected to three-way ANOVA to examine the interactions and post hoc Bonferroni correction to run multiple comparisons and determine the statistical significance of differences (Fig. 3C; fig. S10, B and C; and tables S7 and S8). Three-way ANOVA was performed using the anova_test() function from the rstatix package. Because the original results exhibited heterogeneity of variance, the white.adjust parameter of anova_test() was set to TRUE to correct for heteroscedasticity. This correction consequently makes the sum of squares to be unavailable.

### Connectomics

The input and output connections of TC-LN (v2L36_R) were accessed and retrieved from neuPrint (https://neuprint.janelia.org/) using v1.2 of the dataset. FlyWire (https://flywire.ai/) data for the FAFB dataset [full adult fly brain (*19*)] were generated from a combination of automatic segmentation and human proofreading. The TC-LN in the right hemisphere of the FAFB dataset can be found at FlyWire (*63*) coordinates 113794, 62873, and 1464, with and in the left hemisphere coordinates 148174, 65171, and 1382. The tracing state of these two neurons at the time of writing is permanently associated with IDs 720575940621983979 and 720575940621214975, respectively.

### Study approval

All protocols involving animals were approved by the Institutional Biosafety Committee of Academia Sinica.

## Supplementary Materials

### This PDF file includes:

Supplementary Note Figs. S1 to S13 Tables S2 to S9 References

## Notes

View/request a protocol for this paper from *Bio-protocol*.
