# Supplementary material for: Mating-driven variability in olfactory local interneuron wiring
Source: Sci Adv. 2022 Feb 18;8(7):eabm7723. doi: 10.1126/sciadv.abm7723 (PMC8856614; doi:10.1126/sciadv.abm7723)
Supplement: Supplementary file 1 — Supplementary Note Figs. S1 to S13 Tables S2 to S9 References [file sciadv.abm7723_sm.pdf]

Science Advances
AAAS

Supplementary Materials for

Mating-driven variability in olfactory local interneuron wiring

Ya-Hui Chou*, Chi-Jen Yang, Hao-Wei Huang, Nan-Fu Liou, Michael Raphael Panganiban, David Luginbuhl, Yijie Yin, Istvan Taisz, Liang Liang, Gregory S. X. E. Jefferis, Liqun Luo

*Corresponding author. Email: yhchou@gate.sinica.edu.tw

Published 18 February 2022, *Sci. Adv.* **8**, eabm7723 (2022)
DOI: 10.1126/sciadv.abm7723

The PDF file includes:

Supplementary Note
Figs. S1 to S13
Tables S2 to S9
References

Other Supplementary Material for this manuscript includes the following:

Table S1. Innervation profiles of all TC-LNs.

Supplementary Note

Glomeruli innervated by TC-LNs

The six glomeruli always innervated by TC-LNs are DA1, DC3, DL3, DL4, VA1d and VA1v. The three glomeruli with > 95% innervation frequency are D, DA4m and DA4l. The nine glomeruli with innervation frequency between 10% and 84% are DA2, DA3, DL1, DL5, DM6, VA6, VL2a, VL2p and column.

Glomeruli innervated by TC-LNs of virgin females and mated females

The numbers of innervated glomeruli with innervation frequencies ≥ 1% are 21 and 24 in virgin (n = 823) and mated females (n = 1244) in y^1 w^* background, respectively. Glomerulus DC1, VA5, VA7l are innervated by TC-LNs in mated females only. These glomeruli and their corresponding innervation frequencies are listed below.

(1) virgin females:

D (98.7%), DA1 (100%), DA2 (12.5%), DA3 (46.4%), DA4m (99.4%), DA4l (100%), DC3 (100%), DL1 (40.6%), DL3 (100%), DL4 (99.6%), DL5 (16.3%), DM3 (2.9%), DM6 (95.3%), VA1d (100%), VA1v (100%), VA3 (2.7%), VA6 (68.8%), VL1 (1.5%), VL2a (27.2%), VL2p (12.4%), column (20.3%).

(2) mated females:

D (98.9%), DA1 (99.7%), DA2 (22.6%), DA3 (57.1%), DA4m (99.9%), DA4l (99.9%), DC1 (1.2%), DC3 (100%), DL1 (44.1%), DL3 (100%), DL4 (99.6%), DL5 (18.1%), DM3 (2.9%), DM6 (90.3%), VA1d (100%), VA1v (100%), VA3 (1.2%), VA5 (3.4%), VA6 (77.0%), VA7l (1.3%), VL1 (2.5%), VL2a (36.7%), VL2p (8.8%), column (23.9%).

GRASP signals of TC-LNs

The split GFPs were fused to CD4, which allow the spGFP to distribute along membranes of the expressed neurons. Accordingly, it has been a concern that a part of GRASP signals may be the result of contact between neurites, instead of the reconstructed GFP in the synapse. A single PN has most abundant dendrites in a given glomerulus. However, nearly 70%-80% analyzed ALs of TC-LN and GH146+ PN GRASP virgins or virgin males do not have GRASP signal (Fig. S13C). These results suggest the majority of GRASP signals uncovered in this study were originated from synapses.

trans-Tango

Based on the original design of the *trans*-Tango system, *trans*-Tango flies reared at 25°C revealed less labeled postsynaptic cells than that in flies reared at 18°C with comparable ages. In this study, flies were reared for either 7-day or 8-day before analyzing the signal, TC-LNs in these flies should have enough time to accumulate hGCG::hICAM1::dNRXN1 to trigger signals. Along this line, virgin females carrying TC-LNs tended to have no or weaker *trans*-Tango signals (Fig. S13F and S13G), suggesting the synapses between TC LNs and their postsynaptic neurons may be much weak

in virgins. The brains with scarce postsynaptic neurons also offer the near single cell type images that allow us to identify the morphologies and types of TC-LN postsynaptic neurons.

Possible recruited TC-LN synaptic neurons in mated females

449-QF labels ~60 lateral LNs that have positive GRASP signals with TC-LN, some of which were newly synapsed by TC-LN after mating. Brain myoinhibitory peptides (MIP) are expressed in brain tissue to inhibit food intake (*64*) and to enhance polyamide preference of mated females (*65*). The latter effect is mediated by binding of MIP to its receptor, sex peptide receptor (SPR). Interestingly, both MIP and SPR are expressed in subset(s) of lateral LNs (*57, 60*). In the future, it will be interesting to test whether the lateral LNs newly synapsed to TC-LNs are Mip- and/or SPR-positive LNs. In addition, *trans*-Tango revealed at least one ventral LNs (1702323386) was a postsynaptic target of TC-LNs in mated females. This LN innervates a subset of glomeruli targeted by pheromone-sensing ORNs. It may form an additional inhibitory forward-loop by TC-LN in these pheromone-related glomeruli.

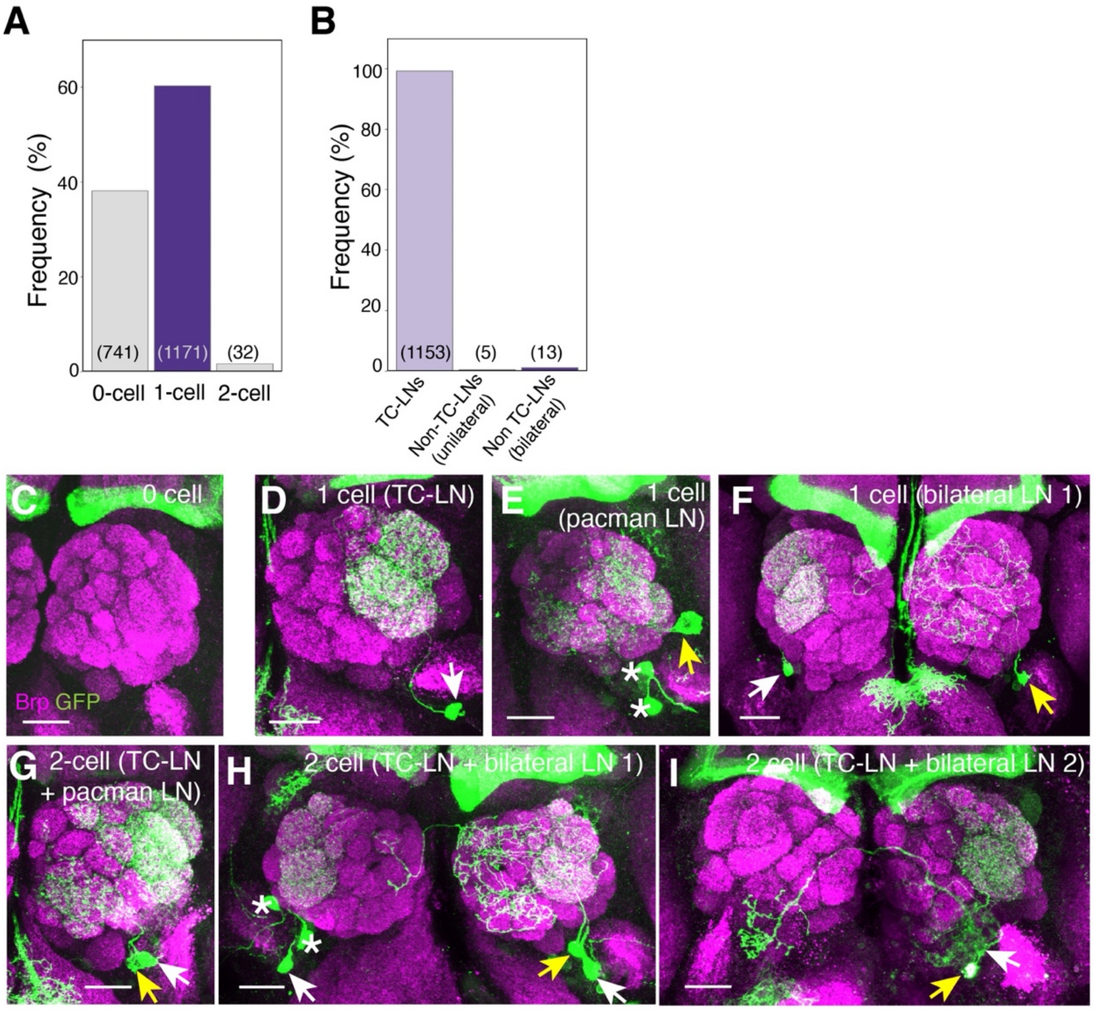

Fig. S1. Frequencies of labeled TC-LNs in *GAD1-GAL80*;*OK107-GAL4* flies.

(**A**) Frequencies of analyzed antennal lobes (n = 1944) that with zero, one, or two labeled LNs (see panel B and G–I for composition). (**B**) Frequencies of labeled LN types in antennal lobes with one labeled LN. (**C**) Representative confocal images of antennal lobes lacking labeled LN. Scale bar, 20 μm. (**D** to **F**) Representative confocal images of a TC-LN (D, white arrow), a single labeled second type of unilateral LN, pacman LN (yellow arrow, named after its innervation pattern, v2LN30) (E), and a bilateral LN (f, yellow arrow). Note that in (F), the contralateral antennal lobe has a labeled TC-LN (white arrow). Asterisks, non-LN neurons that innervate other neuropils than antennal lobes. Scale bars, 20 μm. (**G** to **I**) Two labeled cells in (A) belong to the following three categories: (1) one TC-LN (white arrow) and one pacman LN (yellow arrow) (G), (2) one TC-LN (white arrows) and one bilateral LN (yellow arrow; type 1) (H, right antennal lobe), and (3) one TC-LN (white arrow) and one bilateral LN (yellow arrow, type 2) (I). Asterisks, non-LN neurons that innervate other neuropils than antennal lobes. Scale bars, 20 μm.

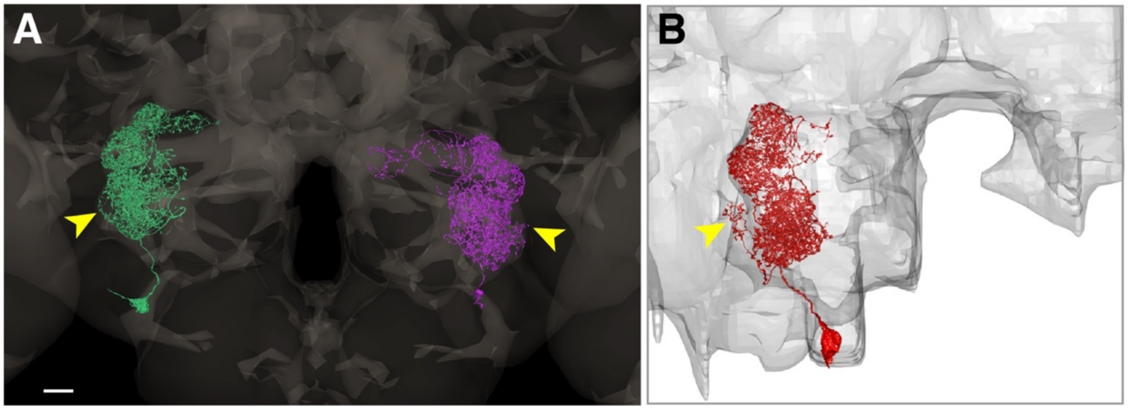

Fig. S2. One TC-LN in each brain hemisphere.
(**A**-**B**) Only one TC-LN was recovered in each brain hemisphere reconstructed from serial EM sections of a whole-brain (A) or a hemi-brain (B). VL2a was innervated by all three TC-LN (yellow arrowheads). (B) is same as Fig. 1C. Scale bar, 15 μm.

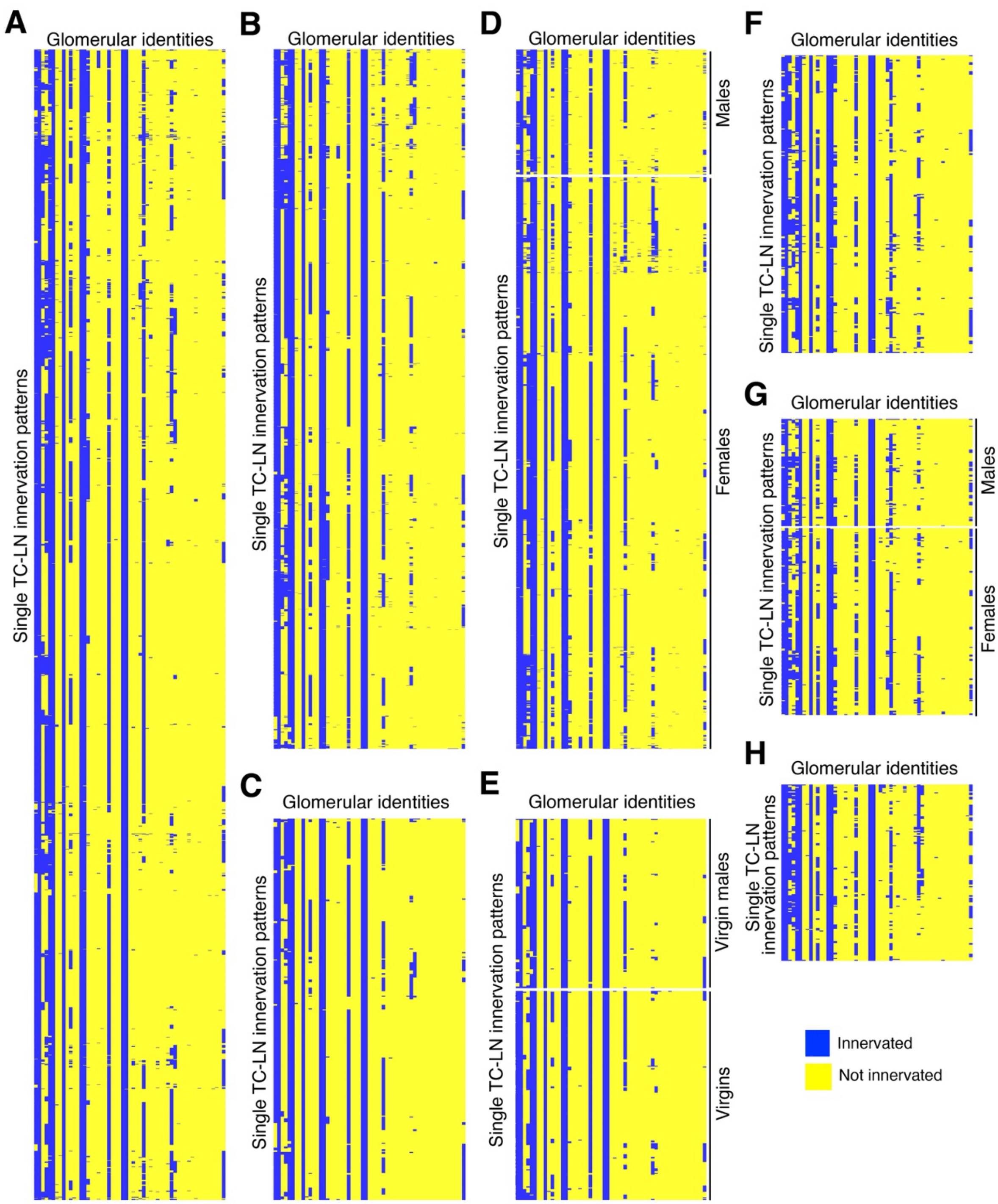

Fig. S3. Hierarchical clustering of the innervation patterns of TC-LNs.
(**A**) All 3300 TC-LNs analyzed in this study. (**B**) 2764 TC-LNs derived from flies with single genetic background y^1 w^*. Same as Fig. 1D. (**C**) 420 TC-LNs derived from newly eclosed virgins and virgin males (0–2-h-old) with single genetic background y^1 w^*. (**D**) 496 and 2268 TC-LNs of male (top) and female (bottom) flies with single genetic background y^1 w^*. (**E**) 188 and 232 TC-LNs of virgin males (top) and virgins (bottom) with single genetic background y^1 w^*. (**F**) 337 TC-

LNs derived from flies with single genetic background *Canton S*. (**G**) 123 and 214 TC-LNs of male and female flies with single genetic background *Canton S*. (**H**) 199 TC-LNs derived from female flies with *fru*$^{+/-}$, *fru*$^{-/-}$, *SPR*$^{+/-}$, *SPR*$^{-/-}$, *Mip*$^{+/-}$ or *Mip*$^{-/-}$.

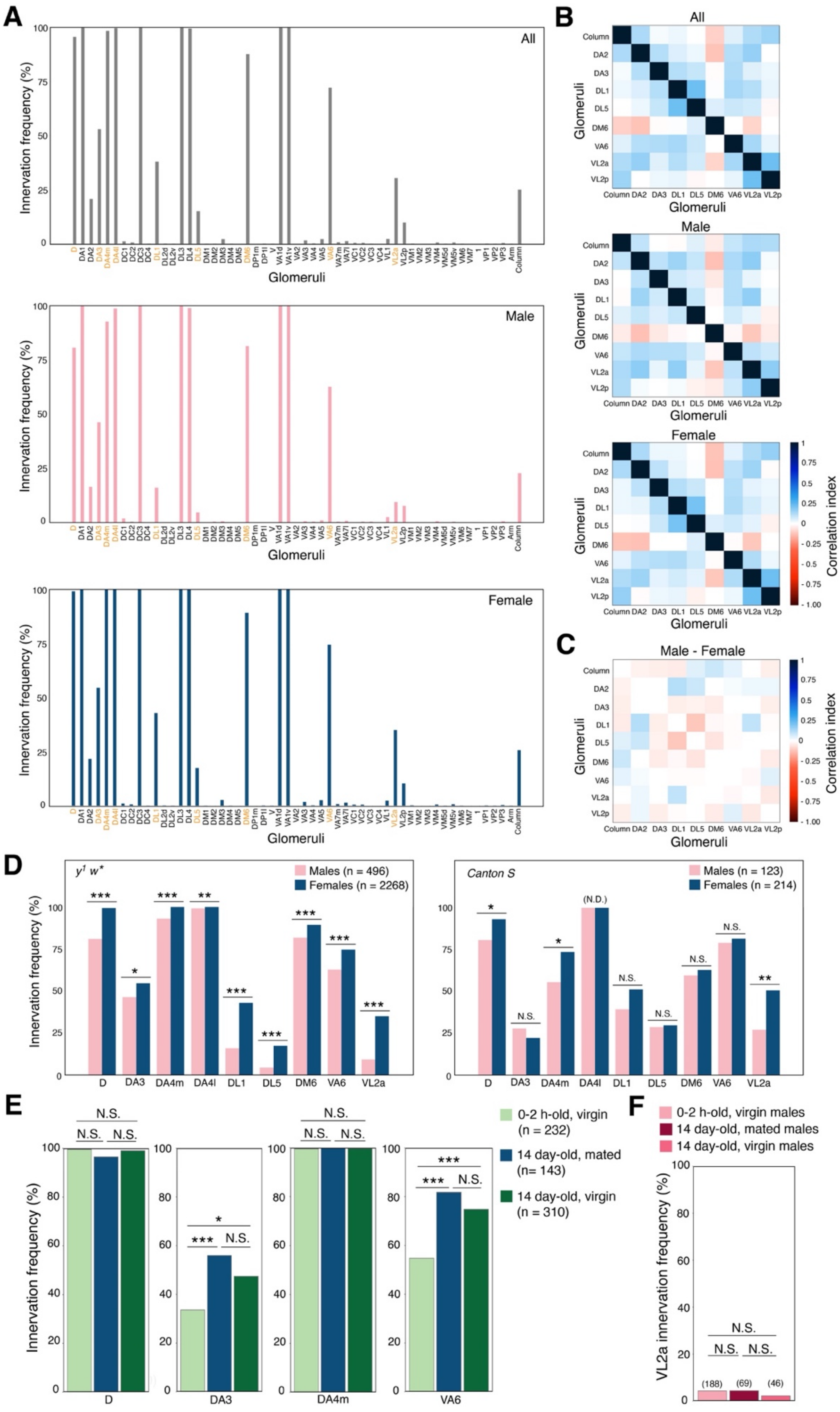

A
All
Male
Female
Innervation frequency (%)
Glomeruli
B
C
Male - Female
Correlation index
D
y1 w*
Males (n = 496)
Females (n = 2268)
Canton S
Males (n = 123)
Females (n = 214)
E
0-2 h-old, virgin (n = 232)
14 day-old, mated (n= 143)
14 day-old, virgin (n = 310)
F
0-2 h-old, virgin males
14 day-old, mated males
14 day-old, virgin males
VL2a innervation frequency (%)

Fig. S4. Glomerular innervation frequencies of TC-LNs of flies on *y^1 w^** single genetic background.

(**A**) Innervation frequencies of individual glomeruli by all 2764 TC-LNs (top), by 496 TC-LNs from males (middle) or by 2268 TC-LNs from females (bottom). The nine glomeruli with differential innervation frequencies between males and females (d) are shown in orange. (**B**) The correlation of innervation frequencies between two glomeruli by all analyzed TC-LNs (top), TC-LNs from males (middle) or TC-LNs from females (bottom) (Table S4). Note that DM6 showed anti-correlation with DA2, VL2a and Column. (**C**) The difference between the correlation of a given glomerular pair in males and females (Table S4). (**D**) Innervation frequencies of TC-LNs of male and female flies with the genetic background y^1 w^* (left) and *Canton S* (right). Glomeruli with 100 % or 0 % innervation in both sexes were excluded. Chi-squared tests, followed by *post hoc* Bonferroni correction, were conducted to examine the differences between males and females in 43 glomeruli and 28 glomeruli in y^1 w^* and *Canton S*, respectively (Table S3). *, $p < 0.05$; **, $p < 0.01$; ***, $p < 0.001$; N.S., not significant. N.D., not done; DA4l is not among the 28 glomeruli of *Canton S*. The Chi-squared tests may be overly sensitive in calling statistical difference in the y^1 w^* group because of the very large numbers. (**E**) Variable glomerular innervations of TC-LNs caused by female courtship experience. The number of examined antennal lobes are 232, 139, and 310 TC-LNs in 0–2h-old virgins, 14-day-old mated females and 14-day-old virgins, respectively. Chi-squared test, followed by Bonferroni correction was conducted to examine the differences between virgins and mated females in 26 glomeruli in y^1 w^* (Table S5, top). *, $p < 0.05$; **, $p < 0.01$; ***, $p < 0.001$; N.S, not significant. (**F**) VL2a innervations of TC-LNs caused by male courtship experience. The number of examined antennal lobes are 188, 69, and 46 TC-LNs in 0–2h-old virgin males, 14-day-old mated males and 14-day-old virgin males, respectively. Chi-squared test, followed by Bonferroni correction was conducted to examine the differences between virgins and mated males in 26 glomeruli in y^1 w^* (Table S5, bottom). Only the data of VL2a were shown. *, $p < 0.05$; **, $p < 0.01$; ***, $p < 0.001$; N.S, not significant.

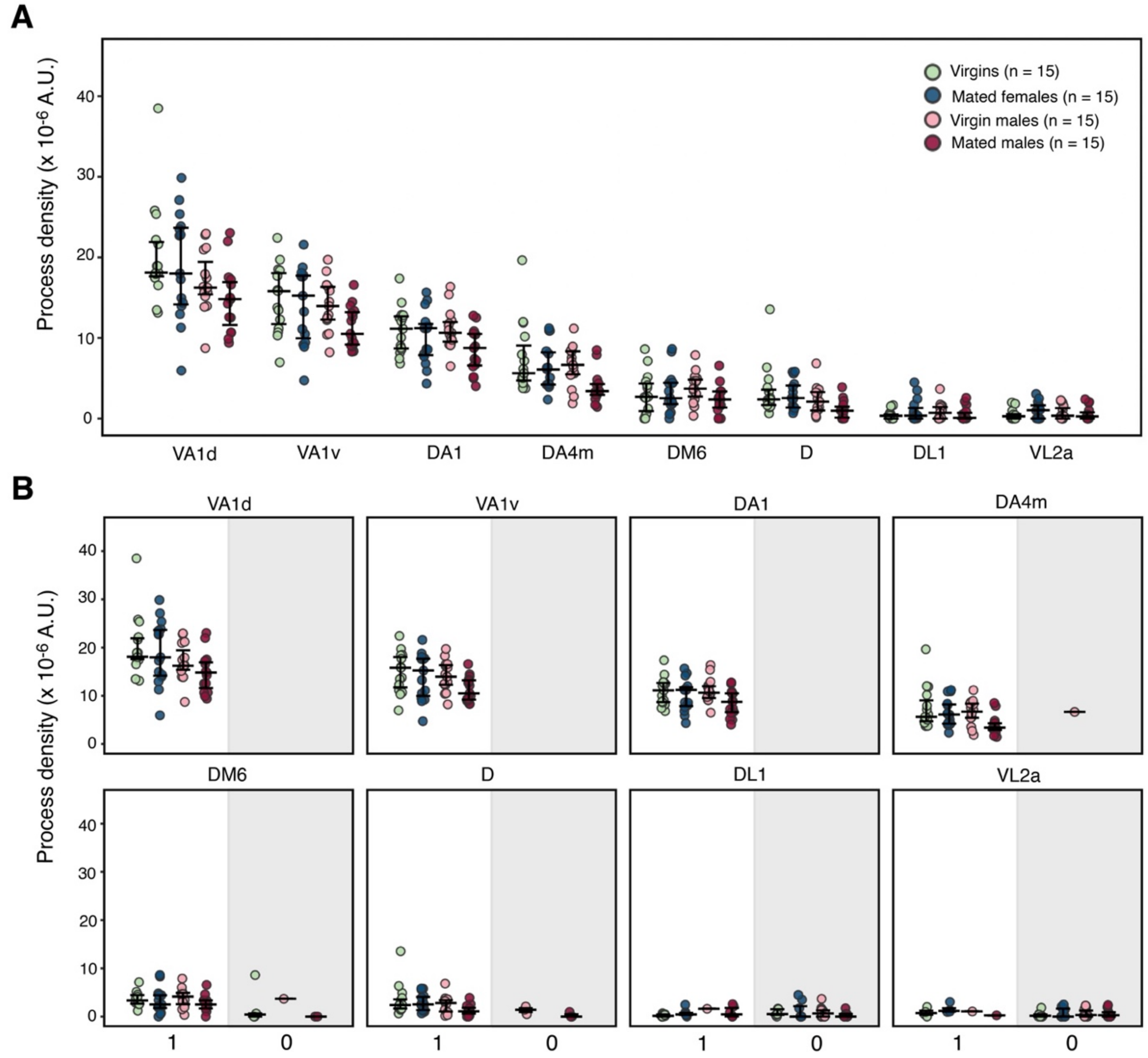

Fig. S5. Variation of TC-LN neurite densities.

(**A**) Process densities of single TC-LNs in VA1d, VA1v, DA1, DA4m, DM6, D, DL1, and VL2a in adult virgins, mated females, virgin males, and mated males. 15 randomly selected cells from each group were examined. See methods for a detailed description of the calculation. Kruskal-Wallis non-parametric one-way ANOVA was used to examine the differences of innervation densities (n = 60) across glomeruli. p = 1.445e-80. *, $p < 0.05$; **, $p < 0.01$; ***, $p < 0.001$; N.S, not significant. (**B**) Process densities of single TC-LNs as in (A) were grouped according to the binary innervation score of the corresponding cells. 1, innervated; 0, not innervated. Note that the density profile lost the power to distinguish true innervation when sparse processes innervated a particular glomerulus (e.g., DL1 and VL2a).

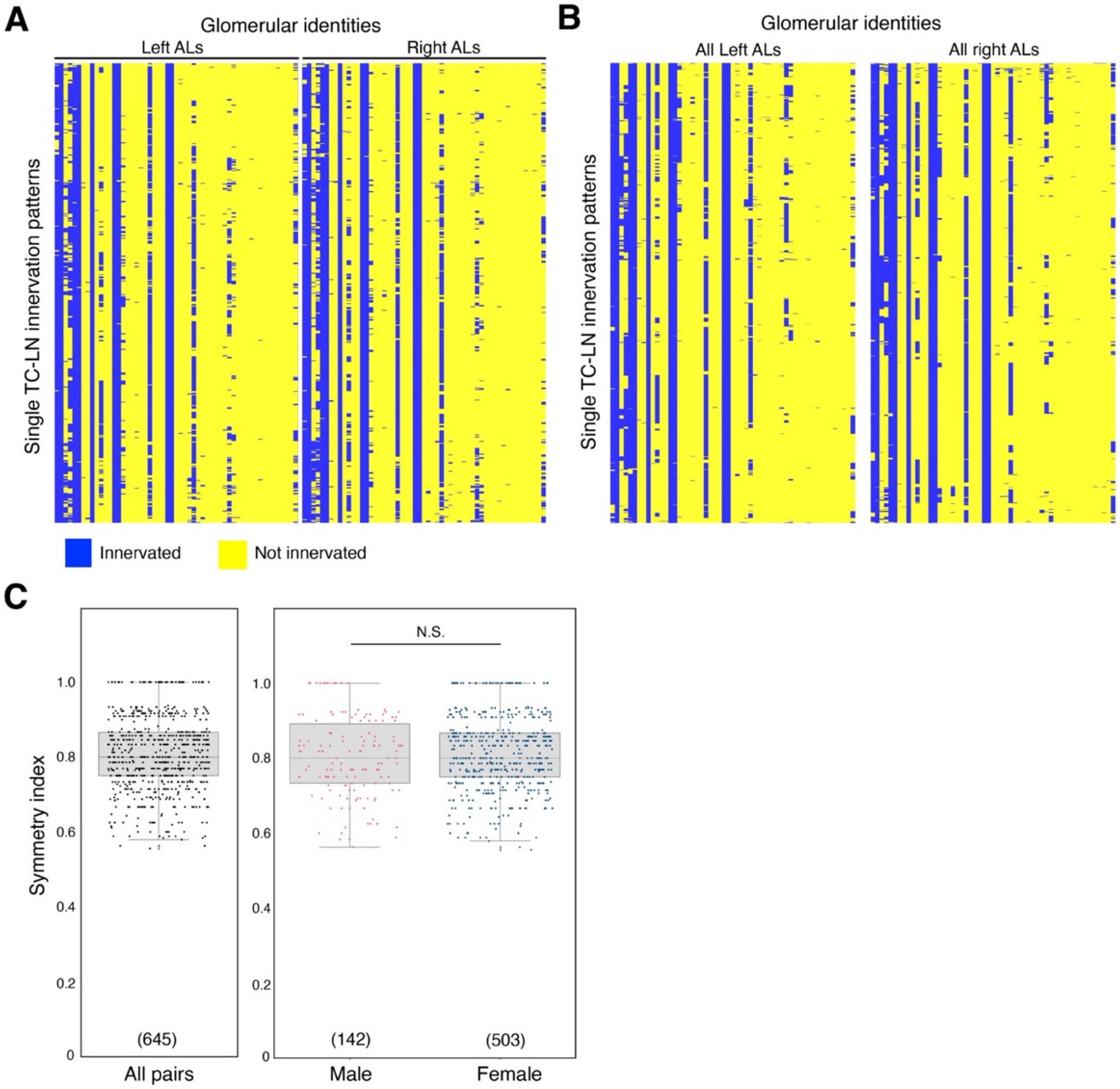

Fig. S6. TC-LNs exhibit differential degrees of intra-individual and inter-individual variability.

(**A**) Hierarchical clustering of paired TC-LNs (n = 645) in the same brains. The left antennal lobe (AL) and right antennal lobe within the same row are from the same fly. (**B**) Hierarchical clustering of all TC-LNs in left antennal lobes (n = 1415) or right antennal lobes (n = 1349). (**C**) Symmetry indices of all TC-LN pairs (left) or TC-LN pairs in male or female brains (right). The grey box indicates the 25-75 percentile range; the range of whiskers indicate 75-percentile + 1.5 interquartile and 25-percentile – 1.5 interquartile. Student's *t*-test was used to compare groups. *, $p < 0.05$; **, $p < 0.01$; ***, $p < 0.001$; N.S., not significant.

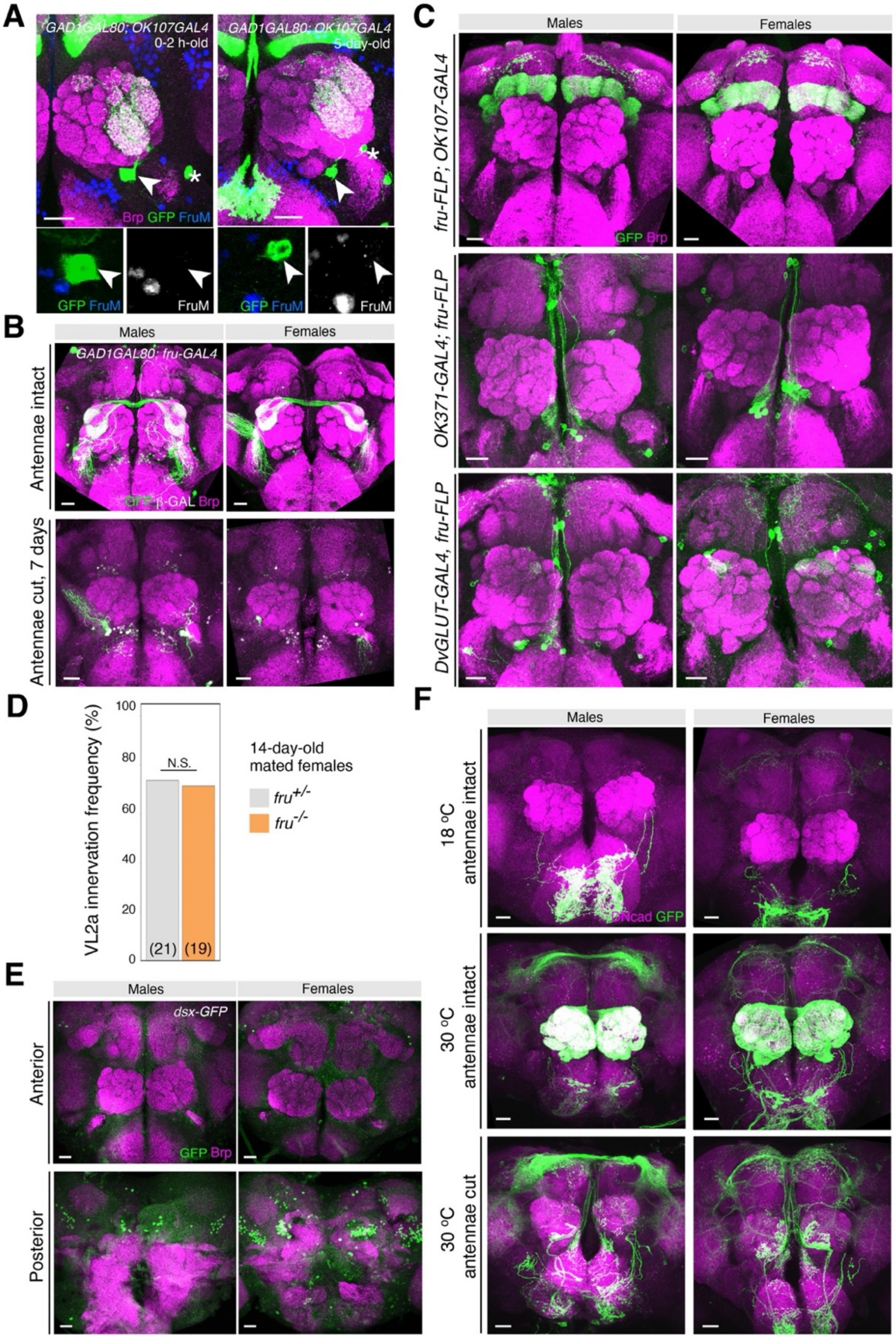

Fig. S7. FruM and Dsx are not expressed in TC-LNs.

(**A**) Fru^{M} is not expressed in TC-LNs. Brains of 0–2-h-old (left) and 5-day-old (right) males were stained for Fru^{M}. Left panels are same as Fig. 2E. Scale bars, 20 μm. (**B**) Confocal images of brains from *GAD1-GAL80, fru-GAL4* flies with intact antennae (top) or antennae cut (bottom). TC-LNs

were not observed in the four analyzed groups. Scale bars, 20 µm. (**C**) *fru-FLP* was used to monitor possible transient expression of Fru^M during development. Because TC-LN is a glutamatergic LN (Fig. S11B), two *GAL4* lines, *OK371-GAL4* that is an enhancer trap of *DvGlut* and *DvGLUT-GAL4*, were also tested. Neither male nor female brains show historical Fru^M expression. Top panels are same as Fig. 2F. Scale bars, 20 µm. (**D**) VL2a innervation by TC-LNs was not affected in *fru* mutant females. Parentheses show the total number of cells examined. Chi-squared test, followed by Bonferroni correction was used to examine the differences between pairs. N.S., not significant. (**E**) Expression of *dsx-GFP* in male and female brains. Note that *dsx-GFP* is only expressed in posterior neurons (posterior sections) and not neurons around the antennal lobe (anterior sections). Scale bars, 20 µm. (**F**) Intersection expression driven by *dsx-GAL4* was used to capture possible transient expressions of *dsx* during development of flies with intact antennae (top, middle) or with antennae cut (bottom). Neither male nor female brains showed historical *dsx* expression in TC-LNs. The bottom images are same as Fig. 2G. Scale bars, 20 µm. Full genotypes were listed in table S9.

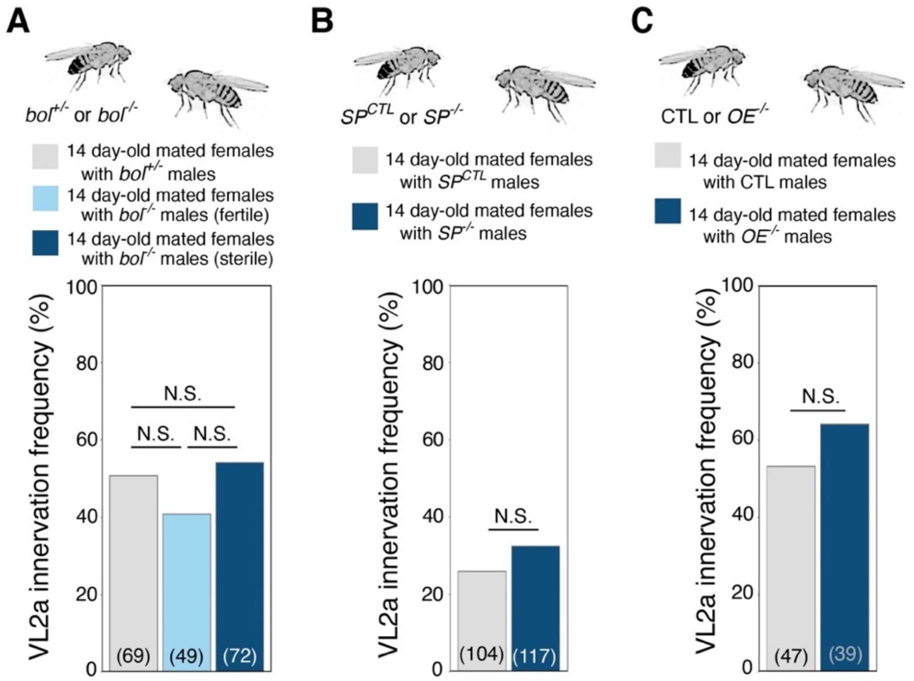

Fig. S8. Sexually dimorphic VL2a innervation by TC-LNs is driven by courtship experience in females.

(**A**-**C**) Removing sperm (A), sex peptide (B) and cuticular hydrocarbons (C) from males did not affect courtship-driven TC-LN innervation in VL2a. *bol* mutant and oenocyte (OE) males fail to produce sperm and cuticular hydrocarbons, respectively. Parentheses show the total number of cells examined. Chi-squared test, followed by Bonferroni correction was used to examine the differences between pairs. N.S., not significant.

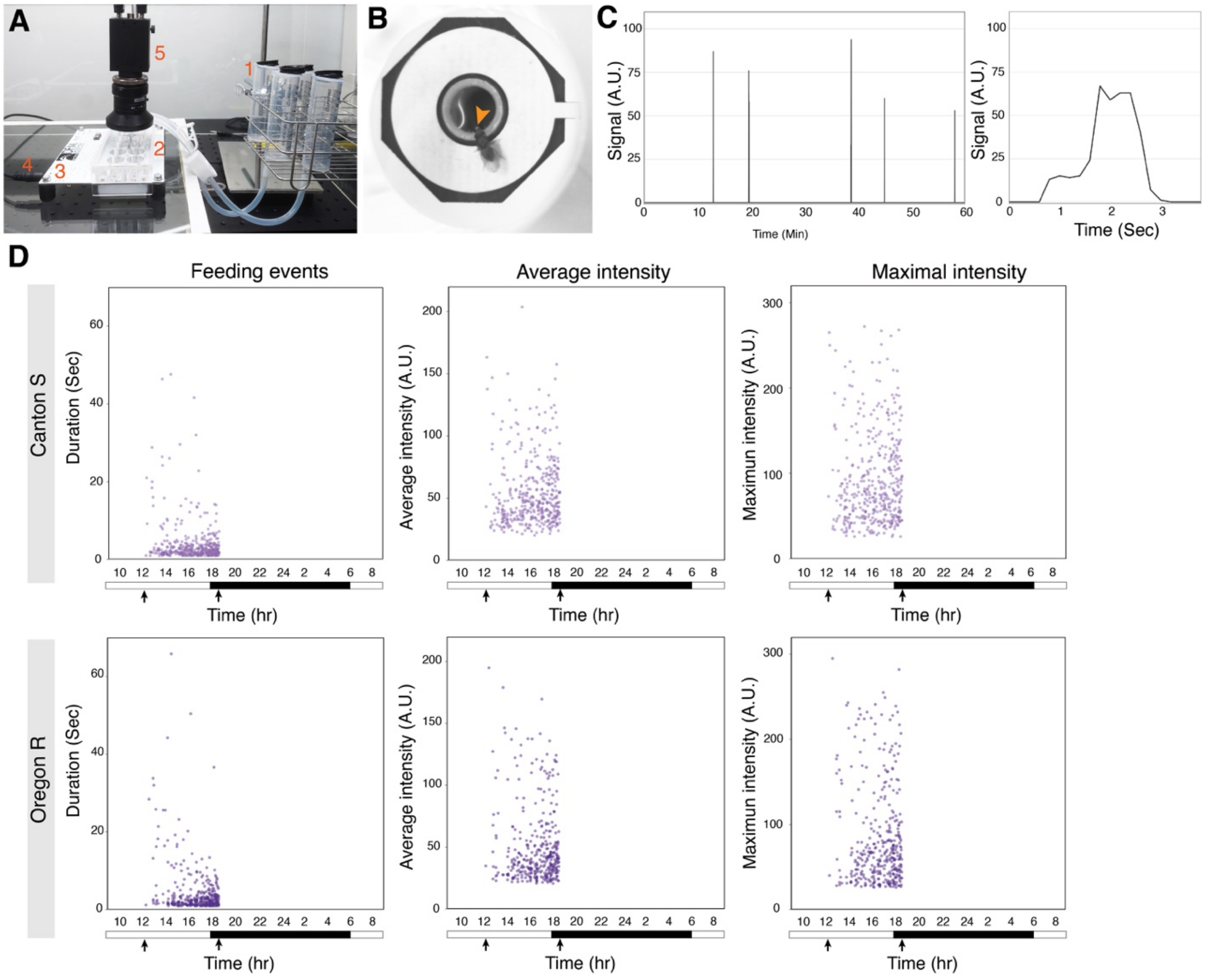

Fig. S9. The Fly Liquid Food Interaction Counter (FLIC) system.
(**A**) The setting of FLIC system. 1, food reservoirs; 2, behavior arena; 3, *Drosophila* feeding monitor (DFM); 4, wire relays signal from DFM to the master control unit (MCU); 5, camera. (**B**, **C**) A fly in one FLIC chamber is shown eating the liquid food in the center through its proboscis (arrowhead) (B); and the FLIC signal (C, left) was reported to the computer in real time. The shape of feeding signals in one feeding event (C, right) is shown. (**D**) Flies on the *Canton S* (n = 12) single genetic background (top panels) and *Oregon R* (n = 12) isogenic background (bottom panels) were subjected to the FLIC system to monitor eating behaviors under light-dark cycle. The feeding events (n = 413 and 375 of *Canton S* and *Oregon R*, respectively) (left), average intensity (middle) and maximal intensity (right) were analyzed and plotted against time. Pairs of arrows indicate the time intervals of FLIC experiments.

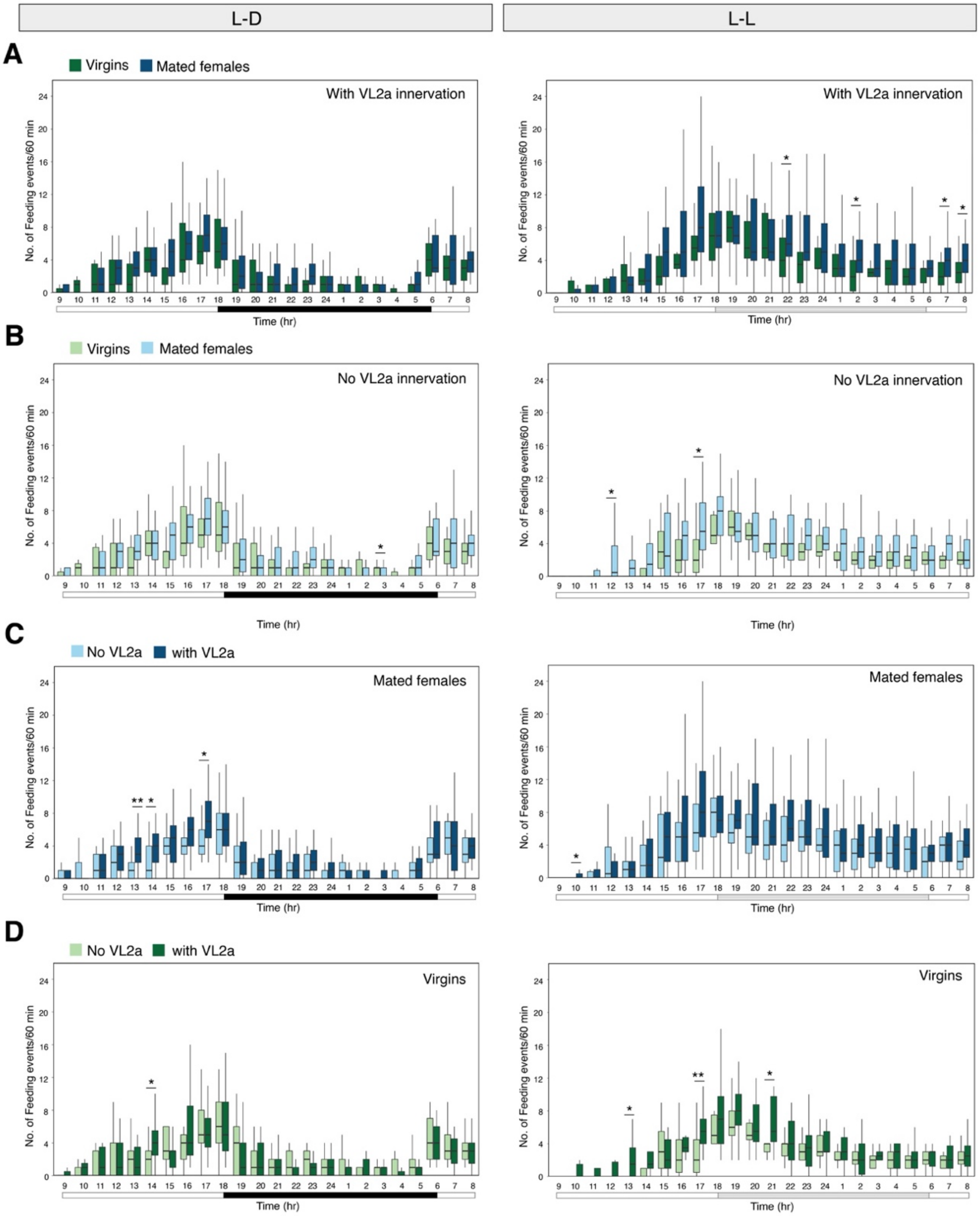

Fig. S10. Feeding events for females and males with or without VL2a innervation by TC-LNs.

(**A**, **B**) Feeding events within 24 h of virgins and females with VL2a innervation (A) or without VL2a innervation (B) under light-dark (L-D) (left panels) or constant light (L-L) (right panels). Hatched bars mark the subjective nights in L-L experiments. (A) is the same as Fig. 3C. (**C**, **D**) Feeding events within 24 h for all mated females (C) or all virgins (D) with VL2a innervation and

without VL2a innervation under light-dark (L-D) (left panels) or constant light (L-L) (right panels). Three-way ANOVA (A-D) was used to examine the main effects and interactions of variables, followed by *post hoc* Bonferroni test to analyze multiple comparisons among different conditions. *$p < 0.05$, **$p < 0.01$, ***$p < 0.001$.

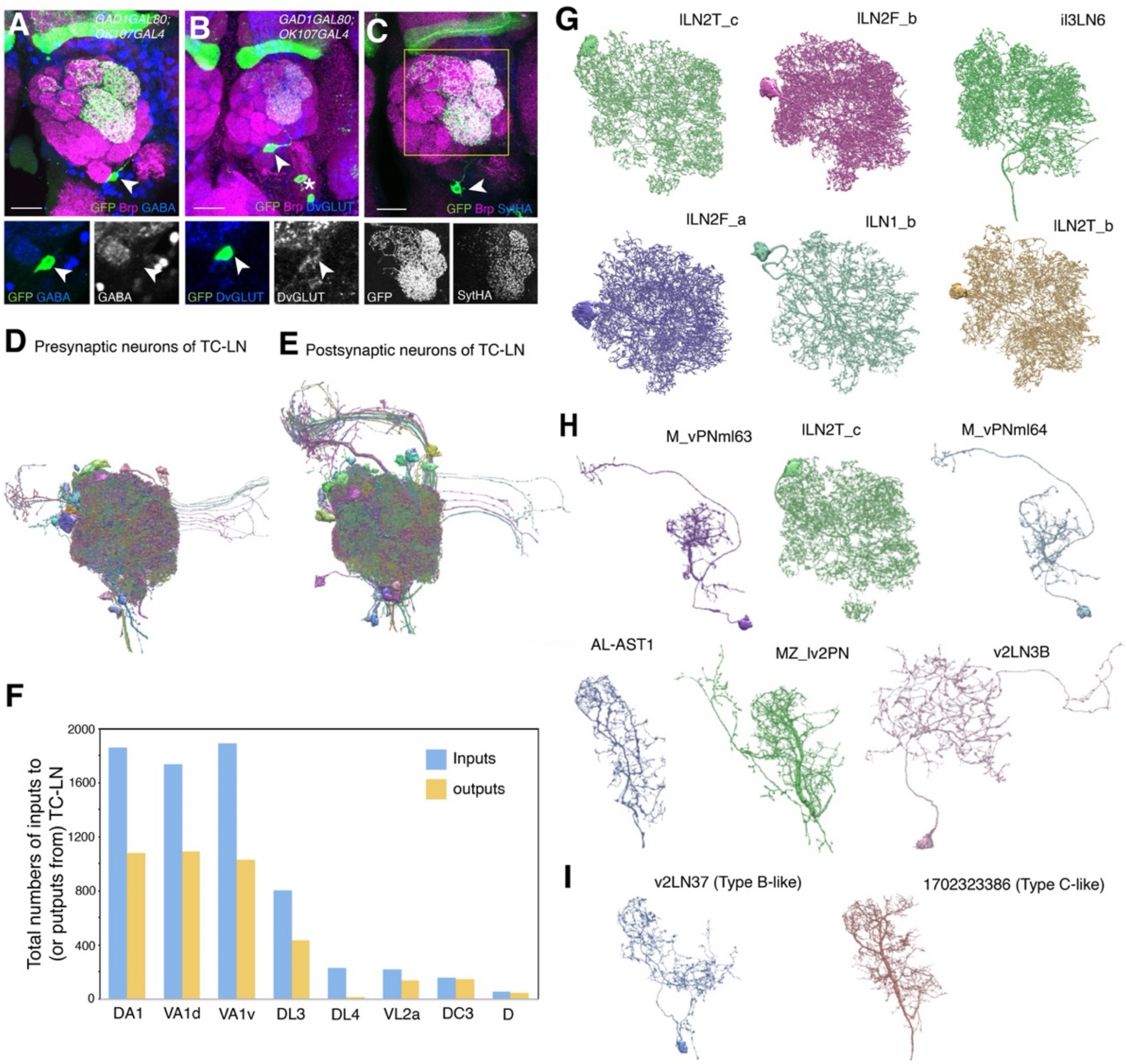

Fig. S11. The local circuits of TC-LNs.

(**A**, **B**) TC-LNs are glutamatergic. Adult brains were stained with GFP (green), neuropil marker (magenta) and GABA (blue in A) or DvGLUT (blue in B). Scale bars, 20 μm. (**C**) Adult brains carrying TC-LNs stained with pre-synaptic marker, SytHA (blue), as well as neuropil marker (magenta) and GFP (green). The presynapses of TC-LNs are roughly evenly distributed along processes, suggesting TC-LNs lack clear axon-dendrite compartmentation. Scale bar, 20 μm. (**D**, **E**) The input neurons (D) and output neurons (E) of TC-LN (v2LN36_R) in the reconstructed hemibrain. v2LN36_R receives 7315 input contacts from 105 types of neurons and have 6310 output contacts to 114 types of neurons. Only neurons with ≥ 0.1 % of total connections with v2LN36_R and one neuron of each type was shown. (**F**) The total numbers of input to and output from v2LN36_R (TC-LN) in individual glomeruli. (**G**, **H**) Top six types of input (F) and output (G) neurons that have most connections with v2L36_R neuron. ORNs and uni-glomerular PNs form majority inputs and outputs to v2L36_R and were not counted here (also see Fig. S4A and 4B). (**I**) Two v2LN36_R output neurons are like Type B and Type C neurons uncovered from TC-LN *trans*-Tango.

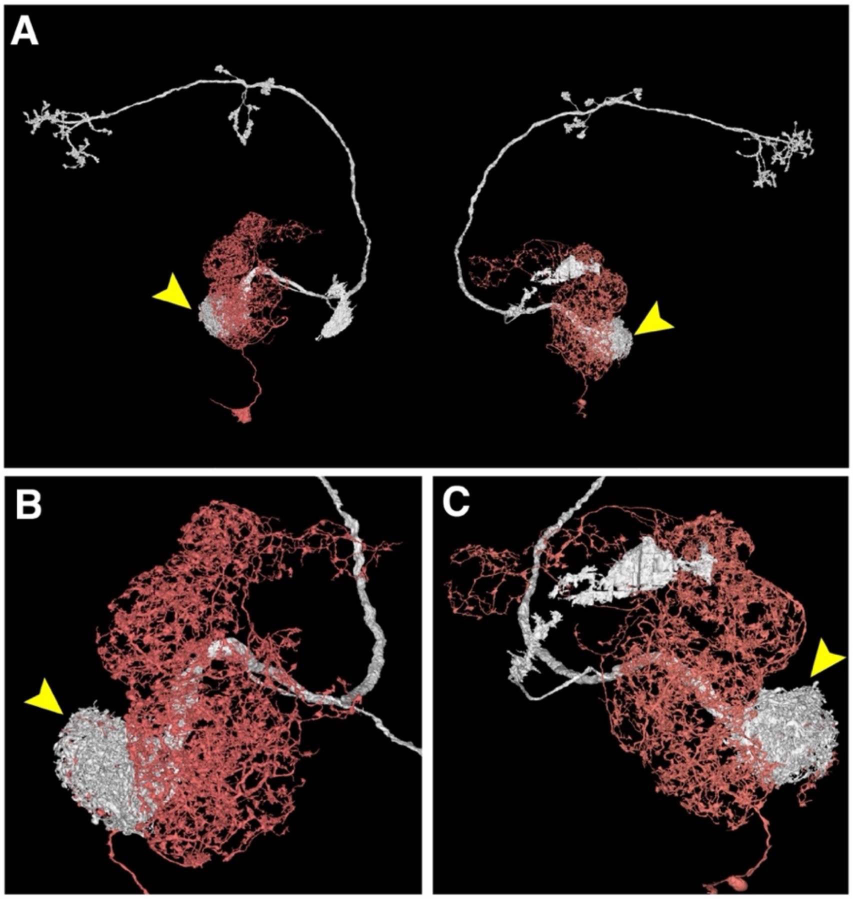

Fig. S12. Reconstructed TC-LNs and VL2a PNs from the FAFB brain.
(**A**-**C**) Reconstructed TC-LNs (red) and VL2a PNs (white) from the FAFB brain (A) showing the synaptic connections between the processes of right TC-LN (v2LN36_R) (B) or left TC-LN (v2LN36_L) (C) and the dendrites of VL2a PNs in the VL2a glomeruli (arrowheads).

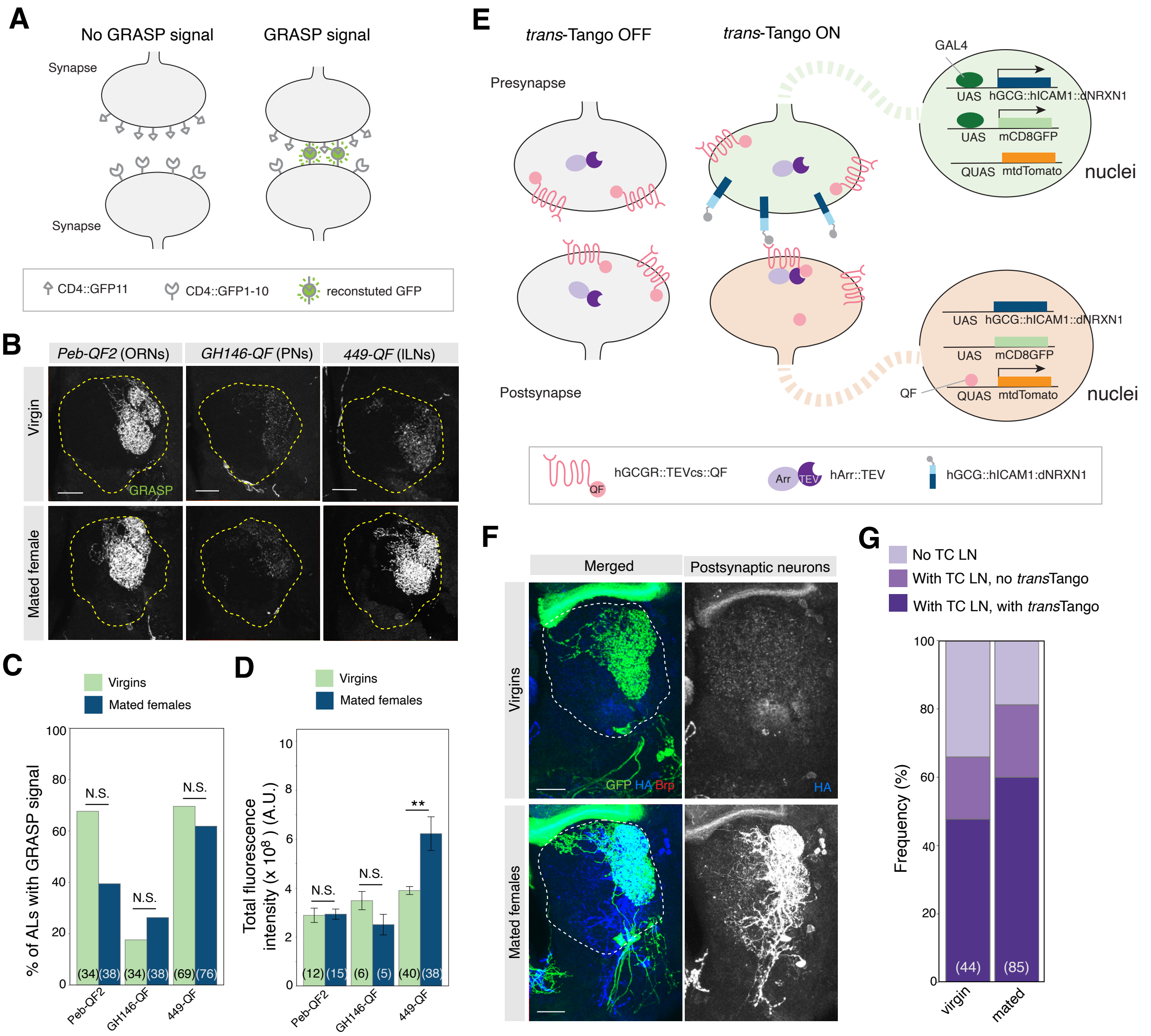

Fig. S13. The synaptic connections of TC-LNs changed in mated females.

(**A**) Schematic diagram of GRASP. GFP is divided into two fragments, the first 11 amino acid (GFP11) and the fragment lacking first 10 amino acids (GFP1-10). These two fragments are fused to membrane protein CD4 and expressed in two different neurons. When these two neurons form synapse (or contact to each other), the two halves of GFP reunite and form a reconstituted GFP. (**B**) GRASP signal (green) between ORNs and TC-LN (left), between PNs and TC-LN (middle) or between lateral unilateral LNs and TC-LNs (right) from 7-day-old females. Scale bars, 20 µm. (B) is the same as Fig. 4D. (**C**) Quantification of ALs with GRASP signal in (B). Parentheses show the total number of TC-LNs examined. cells examined. Chi-squared test, followed by Bonferroni correction was used to examine the differences between pairs. *, $p < 0.05$; **, $p < 0.01$; ***, $p < 0.001$; N.S., not significant. (**D**) Quantification of totally fluorescence intensity in the antennal lobes in (B). Parentheses are numbers of antennal lobes with TC-LN examined. Two-way ANOVA with a *post hoc* Bonferroni correction for multiple comparison was used. Same as Fig. 4E. *, $p <$

0.05; **, $p < 0.01$; ***, $p < 0.001$; N.S., not significant. (**E**) Schematic diagram of *trans*-Tango. The *trans*-Tango system includes three major components, hGCGR::TEVcs::QF (pink), hArr::TEV (magenta) and hGCG::hICAM1::dNRXN1 (blue). The first two components are ubiquitously expressed in all cells. By contrast, the ligand hGCG::hICAM1::dNRXN1 is driven by GAL4. When no GAL4 is expressed in the pre-synapse (left), the *trans*-Tango system is off. When GAL4 is expressed in the presynaptic neurons, it drives the expression of mCD8GFP (green) and hGCG:hICAM1:dNRXN1. The ligand will bind to postsynaptic receptors hGCG:TEVcs:QF (right), which then recruit hArr::TEV to its cytosolic tail, causing the cleavage and release of QF. QF will then binds QUAS and turn on the expression of mtdTomato. In this way, all postsynaptic neurons of a give neuron can be visualized. (After *(43)*.) (**F**) Representative *trans*-Tango signal (HA, blue) from 8-day old females carrying TC-LNs (green). Scale bars, 20 μm. (**G**) Frequencies of ALs without labeled TC-LNs, with labeled TC-LNs but no *trans*-Tango signal, or with both labeled TC-LNs and *trans*-Tango+ cells. No trans-Tango signal was detected in the antennal lobes without labeled TC-LNs. Parentheses indicate the number of antennal lobes examined.
Full genotypes were listed in Table S9.

Table S2. Statistical test results for Figures 1F, 3C, 4E, S5A, S6C and S13D.

Figure	Group[*]	*p*-Value
Fig. 1F[1]	Intra-individual (n = 645) vs. Inter-individual (L-based) (n = 1415)	2.7e-26
	Intra-individual vs. Inter-individual (R-based) (n = 1349)	5.1e-25
	Inter-individual (L-based) vs. Inter-individual (R-based)	0.924
Fig. 3C, top panel[2]	(L-D, no VL2a) virgins (n = 17) vs. mated females (n = 17)	5.46e-01
	(L-D, with VL2a) virgins (n = 15) vs. mated females (n = 19)	3.00e-03
	(L-L, no VL2a) virgins (n = 15) vs. mated females (n = 14)	3.81e-06
	(L-L, with VL2a) virgins (n = 14) vs. mated females (n = 19)	8.83e-07
	(no VL2a) (L-L) virgins vs. (L-D) virgins	7.10e-02
	(no VL2a) (L-L) mated females vs. (L-D) mated females	3.04e-10
	(with VL2a) (L-L) virgins vs. (L-D) virgins	1.69e-08
	(with VL2a) (L-L) mated females vs. (L-D) mated females	3.20e-14
Fig. 3C, bottom panel[2]	(L-D virgins) No VL2a vs. VL2a	0.874
	(L-D mated females) No VL2a vs. VL2a	0.000431
	(L-L virgins) No VL2a vs. VL2a	0.0000112
	(L-L mated females) No VL2a vs. VL2a	0.0000220
Fig. 4E, Fig. S13D[3]	*Peb-QF2* GRASP, virgins (n = 12) vs. mated females (n = 15)	0.899
	GH146-QF GRASP, virgins (n = 6) vs. mated females (n = 5)	0.15
	449-QF GRASP, virgins (n = 40) vs. mated females (n = 38)	0.003
Fig. S5A[4]	VA1d (n = 60), VA1v (n = 60), DA1 (n = 60), DA4m (n = 60), DM6 (n = 60), D (n = 60), DL1 (n. = 60), VL2a (n = 60)	1.445e-80
Fig. S6C[5]	males (n = 142) vs. females (n = 503)	0.4487

[*] N, number of trials; n, total number of cells or ALs.
[1] Wilcoxon rank sum test.
[2] Three-way ANOVA for examining main effects and interactions.
[3] Two-way ANOVA with a *post hoc* Bonferroni correction for multiple comparisons.
[4] Kruskal-Wallis non-parametric one-way ANOVA.
[5] Student's *t*-test.

Table S3. Chi-squared test of glomeruli exhibiting sexual dimorphic TC-LN innervations in Figures 2A, 2B and S4D.

Genetic background: y^1 *w**				Genetic background: *Canton S*			
Glomerulus	X-squared	df	*p* -value[1]	Glomerulus	X-squared	df	*p* -value[1]
D	323.68	1	< 2.2e-16	D	10.741	1	0.029344
DA1	0.003333	1	1	DA2	0.067853	1	1
DA2	6.694	1	0.415982	DA3	1.0864	1	1
DA3	10.881	1	0.0417745	DA4m	10.706	1	0.029904
DA4m	128.39	1	< 2.2e-16	DC1	4.95E-30	1	1
DA4l	17.518	1	0.00122421	DC3	7.82E-32	1	1
DC1	1.0252	1	1	DL1	3.9868	1	1
DC2	0.64654	1	1	DL2d	2.86E+00	1	1
DC4	1.25E-25	1	1	DL4	4.55E-29	1	1
DL1	123.38	1	< 2.2e-16	DL5	0.004477	1	1
DL2d	0.003333	1	1	DM3	0.23807	1	1
DL2v	1.25E-25	1	1	DM6	0.2275	1	1
DL4	3.13	1	1	VA1v	7.82E-32	1	1
DL5	52.267	1	2.08335E-11	VA3	0.092476	1	1
DM1	8.32E-30	1	1	VA5	1.91E-01	1	1
DM2	0.69807	1	1	VA6	0.16184	1	1
DM3	8.122	1	0.188039	VA7m	5.3863	1	0.5684
DM4	8.32E-30	1	1	VA7l	0.32138	1	1
DM6	21.169	1	0.000180772	VC1	0.078885	1	1
DP1l	0.003333	1	1	VC2	1.29E+00	1	1
V	8.32E-30	1	1	VL1	4.71E-31	1	1
VA3	4.1656	1	1	VL2a	1.70E+01	1	0.00105952
VA4	1.63E-26	1	1	VL2p	4.20E-30	1	1
VA5	4.731	1	1	VM4	0.078885	1	1
VA6	27.841	1	5.6631E-06	VM5v	0.092476	1	1
VA7m	1.3124	1	1	VM6	7.82E-32	1	1
VA7l	1.3685	1	1	VP2	7.82E-32	1	1
VC1	1.3467	1	1	Column	1.1068	1	1
VC2	0.24267	1	1				
VC3	0.69807	1	1				
VL1	1.13E-29	1	1				
VL2a	125.5	1	< 2.2e-16				
VL2p	3.4424	1	1				
VM1	0.080656	1	1				
VM4	0.016738	1	1				
VM5d	1.25E-25	1	1				
VM5v	0.8027	1	1				
VM6	8.32E-30	1	1				
VP1	0.003333	1	1				
VP2	8.32E-30	1	1				
VP3	1.44E-29	1	1				
Arm	1.25E-25	1	1				
Column	1.7858	1	1				

[1] *Post hoc* Bonferroni correction was used for multiple comparison. Only adjusted p-values were shown.

Table S4. Correlation of glomerular pairs of all TC-LNs in Figure S4B and differences between the correlation of glomerular pairs of TC-LNs in males and females in Figure S4C.

Figure S4B, top

	Column	DA2	DA3	DL1	DL5	DM6	VA6	VL2a	VL2p
Column	1	0.127383	0.02253856	0.02817291	0.00665522	-0.1013927	0.0545844	0.12209241	0.15488353
DA2	0.127383	1	0.11098954	0.06057168	0.02475197	-0.1213726	0.11550452	0.16044956	-0.0032509
DA3	0.02253856	0.11098954	1	0.11558462	0.08850516	-0.0086361	0.12101683	0.05383882	0.02793459
DL1	0.02817291	0.06057168	0.11558462	1	0.21918073	0.01370499	0.13393953	0.11672408	0.03798118
DL5	0.00665522	0.02475197	0.08850516	0.21918073	1	0.0621427	0.08490675	0.06796836	-0.0196911
DM6	-0.1013927	-0.1213726	-0.0086361	0.01370499	0.0621427	1	-0.0060783	-0.0860772	-0.0024605
VA6	0.0545844	0.11550452	0.12101683	0.13393953	0.08490675	-0.0060783	1	0.13503411	0.03883777
VL2a	0.12209241	0.16044956	0.05383882	0.11672408	0.06796836	-0.0860772	0.13503411	1	0.221911
VL2p	0.15488353	-0.0032509	0.02793459	0.03798118	-0.0196911	-0.0024605	0.03883777	0.221911	1

Figure S4B, middle

	Column	DA2	DA3	DL1	DL5	DM6	VA6	VL2a	VL2p
Column	1	0.10057191	-0.0068643	-0.0110519	0.04759764	-0.0400185	0.08964215	0.12655293	0.12195662
DA2	0.10057191	1	0.10506079	0.15052301	0.09026505	-0.1259517	0.12815313	0.1784022	0.01988016
DA3	-0.0068643	0.10506079	1	0.07212532	0.05583998	-0.0470722	0.11590548	0.03850898	-0.0012723
DL1	-0.0110519	0.15052301	0.07212532	1	0.093555	-0.0190902	0.10953768	0.16474174	0.02321045
DL5	0.04759764	0.09026505	0.05583998	0.093555	1	0.00203177	0.06573962	0.03239823	-0.0238957
DM6	-0.0400185	-0.1259517	-0.0470722	-0.0190902	0.00203177	1	-0.0160713	-0.115655	-0.0421904
VA6	0.08964215	0.12815313	0.11590548	0.10953768	0.06573962	-0.0160713	1	0.10408717	0.07727015
VL2a	0.12655293	0.1784022	0.03850898	0.16474174	0.03239823	-0.115655	0.10408717	1	0.17376356
VL2p	0.12195662	0.01988016	-0.0012723	0.02321045	-0.0238957	-0.0421904	0.07727015	0.17376356	1

Figure S4B, bottom

	Column	DA2	DA3	DL1	DL5	DM6	VA6	VL2a	VL2p
Column	1	0.13112614	0.02672886	0.0284763	-0.002023	-0.1216972	0.04358537	0.1196962	0.16000533
DA2	0.13112614	1	0.10884821	0.03679569	0.01046306	-0.1279932	0.10784372	0.15167424	-0.0093573
DA3	0.02672886	0.10884821	1	0.11059824	0.08518702	-0.0055489	0.11537685	0.04221396	0.03079184
DL1	0.0284763	0.03679569	0.11059824	1	0.2058007	-0.0026643	0.11817434	0.06593524	0.03199516
DL5	-0.002023	0.01046306	0.08518702	0.2058007	1	0.05997535	0.07488867	0.04006677	-0.0254449
DM6	-0.1216972	-0.1279932	-0.0055489	-0.0026643	0.05997535	1	-0.0150103	-0.1108334	0.00272784
VA6	0.04358537	0.10784372	0.11537685	0.11817434	0.07488867	-0.0150103	1	0.12120825	0.02670772
VL2a	0.1196962	0.15167424	0.04221396	0.06593524	0.04006677	-0.1108334	0.12120825	1	0.22587033
VL2p	0.16000533	-0.0093573	0.03079184	0.03199516	-0.0254449	0.00272784	0.02670772	0.22587033	1

Figure S4C

	Column	DA2	DA3	DL1	DL5	DM6	VA6	VL2a	VL2p
Column	0	-0.0305542	-0.0335931	-0.0395282	0.04962064	0.0816787	0.04605678	0.00685672	-0.0380487
DA2	-0.0305542	0	-0.0037874	0.11372733	0.079802	0.00204155	0.0203094	0.02672795	0.02923744
DA3	-0.0335931	-0.0037874	0	-0.0384729	-0.029347	-0.0415233	0.00052863	-0.003705	-0.0320642
DL1	-0.0395282	0.11372733	-0.0384729	0	-0.1122457	-0.0164258	-0.0086367	0.09880651	-0.0087847
DL5	0.04962064	0.079802	-0.029347	-0.1122457	0	-0.0579436	-0.0091491	-0.0076685	0.00154919
DM6	0.0816787	0.00204155	-0.0415233	-0.0164258	-0.0579436	0	-0.001061	-0.0048217	-0.0449183
VA6	0.04605678	0.0203094	0.00052863	-0.0086367	-0.0091491	-0.001061	0	-0.0171211	0.05056244
VL2a	0.00685672	0.02672795	-0.003705	0.09880651	-0.0076685	-0.0048217	-0.0171211	0	-0.0521068
VL2p	-0.0380487	0.02923744	-0.0320642	-0.0087847	0.00154919	-0.0449183	0.05056244	-0.0521068	0

Table S5. Chi-squared test of mating effect on glomeruli exhibiting variable TC-LN innervations in Figures 2D, S4E1 and S4F1.

-2-h virgin females (n = 232) v.s. mated females (n = 143)				14-day virgin females (n = 310) v.s. mated females				0-2-h virgin females v.s. 14-day virgin females			
Glomerulus	X-squared	df	p-value[2]	Glomerulus	X-squared	df	p-value[2]	Glomerulus	X-squared	df	p-value[2]
D	3.5129	1	1	D	2.2967	1	1	D	0.046313	1	1
DA2	1.3271	1	1	DA2	0.37965	1	1	DA2	0.32742	1	1
DA3	17.178	1	0.00088478	DA3	2.5138	1	1	DA3	9.8454	1	0.044278
DA4m	1.46E-30	1	1	DA4m	1.29E-32	1	1	DA4m	1.61E-30	1	1
DC1	1.1584	1	1	DC1	1.7542	1	1	DC1	NA	1	NA
DC2	0.059849	1	1	DC2	0.15763	1	1	DC2	NA	1	NA
DC4	1.46E-30	1	1	DC4	NA	1	NA	DC4	0.021188	1	1
DL1	8.3081	1	0.102622	DL1	0.041038	1	1	DL1	14.999	1	0.002795
DL2d	NA	1	NA	DL2d	1.29E-32	1	1	DL2d	1.49E-33	1	1
DL5	6.4673	1	0.28574	DL5	9.1057	1	0.066248	DL5	39.367	1	9.1312E-09
DM3	2.0637	1	1	DM3	0.39839	1	1	DM3	0.55353	1	1
DM6	0.017693	1	1	DM6	0.065752	1	1	DM6	1.3366	1	1
DP1l	NA	1	NA	DP1l	1.29E-32	1	1	DP1l	1.49E-33	1	1
VA3	0.30248	1	1	VA3	0.48689	1	1	VA3	3.6533	1	1
VA4	NA	1	NA	VA4	1.29E-32	1	1	VA4	1.49E-33	1	1
VA5	0.059849	1	1	VA5	2.48E-30	1	1	VA5	1.49E-33	1	1
VA6	27.357	1	4.3992E-06	VA6	2.315	1	1	VA6	23.075	1	0.000040508
VA7m	0.059849	1	1	VA7m	2.48E-30	1	1	VA7m	1.49E-33	1	1
VC2	NA	1	NA	VC2	0.3104	1	1	VC2	0.84182	1	1
VL1	4.9443	1	0.68068	VL1	1.1613	1	1	VL1	1.3235	1	1
VL2a	18.074	1	0.0005525	VL2a	11.571	1	0.01742	VL2a	1.3609	1	1
VL2p	0.047839	1	1	VL2p	0.0001085	1	1	VL2p	0.018006	1	1
VM1	4.03E-31	1	1	VM1	0.15763	1	1	VM1	0.021188	1	1
VM4	4.03E-31	1	1	VM4	2.48E-30	1	1	VM4	1.61E-30	1	1
VP3	NA	1	NA	VP3	1.29E-32	1	1	VP3	1.49E-33	1	1
Column	0.00018747	1	1	Column	0.050284	1	1	Column	0.24032	1	1

0-2h virgin males (n = 188) v.s. mated males (n = 69)				14-day virgin males (n = 46) v.s. mated males				0-2-h virgin males v.s. 14-day virgin males			
Glomerulus	X-squared	df	p-value[2]	Glomerulus	X-squared	df	p-value[2]	Glomerulus	X-squared	df	p-value[2]
D	3.7726	1	1	D	0.11891	1	1	D	3.8051	1	1
DA2	0.94286	1	1	DA2	6.6261	1	0.21105	DA2	5.5903	1	1
DA3	0.07312	1	1	DA3	16.778	1	8.82E-04	DA3	6.5659	1	1
DA4m	13.713	1	0.004473	DA4m	0.36323	1	1	DA4m	11.256	1	0.0166656
DA4l	4.7293	1	0.62265	DA4l	0.82841	1	1	DA4l	0.2178	1	1
DC1	7.19E-31	1	1	DC1	6.85E-29	1	1	DC1	9.42E-30	1	1
DL1	0.0097524	1	1	DL1	7.8051	1	0.10941	DL1	2.0414	1	1
DL4	7.19E-31	1	1	DL4	6.90E-28	1	1	DL4	NA	1	NA
DL5	0.30175	1	1	DL5	1.10E-29	1	1	DL5	0.19082	1	1
DM2	7.19E-31	1	1	DM2	6.90E-28	1	1	DM2	NA	1	NA
DM3	NA	1	NA	DM3	0.27398	1	1	DM3	9.42E-30	1	1
DM6	1.6398	1	1	DM6	3.95E-30	1	1	DM6	0.83121	1	1
VA4	NA	1	NA	VA4	0.27398	1	1	VA4	9.42E-30	1	1
VA6	0.65983	1	1	VA6	4.80E+00	1	0.02851	VA6	5.8767	1	0.32214
VA7l	7.19E-31	1	1	VA7l	6.90E-28	1	1	VA7l	NA	1	NA
VC3	NA	1	NA	VC3	0.27398	1	1	VC3	9.42E-30	1	1
VL1	NA	1	NA	VL1	2.3796	1	1	VL1	0.19082	1	1
VL2a	0.053034	1	1	VL2a	1.12E-30	1	1	VL2a	0.010792	1	1
VL2p	0.053442	1	1	VL2p	0.10021	1	1	VL2p	0.31057	1	1
VM5V	0.58541	1	1	VM5V	NA	1	NA	VM5V	0.042032	1	1
Column	1.4794	1	1	Column	10.525	1	0.024738	Column	1.8471	1	1

[1] Glomeruli exhibiting 100 % or 0% innervation in all three groups were not analyzed.

[2] *Post hoc* Bonferroni correction was used for multiple comparison. Only adjusted p-values were shown. N.A., not available.

Table S6. Chi-squared test for Figures 4G, S7D, S8A-C and S13C.

Figure	X-squared	Degree of freedom	*p*-Value[1]	*p*-Value after correction[2]
Fig. 4G<br>virgin (n = 21) vs. mated female (n = 51)				
Type A	0.48403	1	0.4866	1
Type B	4.26E+00	1	0.03893	0.15572
Type C	15.253	1	9.40e-05	3.76e-04
Type D	0.017287	1	0.017287	1
Fig. S7D				
fru$^{+/-}$ (n =19) v.s. *fru*$^{-/-}$ (n = 21)				
	0	1	1	1
Fig. S8A<br>females with *bol*$^{+/-}$ males (n = 69) v.s. *bol*$^{-/}$- fertile males (n = 49)				
	0.057818	1	0.81	1
females with *bol*$^{+}$/- males (n = 69) v.s. *bol*$^{-/-}$ sterile males (n = 72)				
	0.76728	1	0.3811	1
females with *bol*$^{-/-}$ fertile males (n = 49) v.s. *bol*$^{-/-}$ sterile males (n = 72)				
	1.5799	1	0.2088	1
Fig. S8B				
females with *SP*^{CTL} males (n = 104) v.s. females with *SP*$^{-/-}$ males (n = 117)				
	0.83433	1	0.361	1
Fig. S8C				
females with control males (n = 47) v.s. females with *OE*$^{-/-}$ males (n = 39)				
	0.64248	1	0.4228	1
Fig. S13C				
Peb-QF2 GRASP, virgin (n = 34) vs. mated female (n = 38)				
	4.6404	1	0.03123	0.09369
GH146-QF GRASP, virgin (n = 34) vs. mated female (n = 38)				
	0.35924	1	0.5489	1
449-QF GRASP, virgin (n = 69) vs. mated female (n = 76)				
	0.64357	1	0.4224	1

[1] Chi-squared test.

[2] *Post hoc* Bonferroni correction was used for multiple comparison.

Table S7. Statistical test results for Figures 3B.

Figure	Effects of variables	Degree of freedom	Sum of square	*p*-Value
Two-way ANOVA[1]				
Variables: mating status and time (L-D)				
	Mating status	1	207	2.46e-08
	Time	23	12989	< 2e-16
	Mating status : Time	23	202	0.137
	Residuals	4656	30862	
Variables: mating status and time (L-L)				
	Mating status	1	738	< 2e-16
	Time	23	16456	< 2e-16
	Mating status : Time	23	303	0.142
	Residuals	4824	48195	
Three-way ANOVA[2]				
Variables: mating status, time and VL2a innervation (VL2a) (L-D)				
	Mating status	1	N.A[3,4]	7.60e-02
	Time	23	N.A	8.03e-113
	VL2a	1	N.A	1.08e-02
	Mating status : Time	23	N.A	9.16e-01
	Mating status : VL2a	1	N.A	1.00e-02
	Time:VL2a	23	N.A	2.79e-01
	Mating status : Time : VL2a	23	N.A	6.91e-01
Variables: mating status, time and VL2a innervation (VL2a) (L-L)				
	Mating status	1	N.A	1.83e-11
	Time	23	N.A	5.93e-147
	VL2a	1	N.A	2.26e-09
	Mating status : Time	23	N.A	3.95e-04
	Mating status:VL2a	1	N.A	2.91e-01
	Time:VL2a	23	N.A	2.29e-01
	Mating status : Time : VL2a	23	N.A	7.08e-01

[1] Two-way ANOVA with a *post hoc* Bonferroni correction for multiple comparisons.

[2] Three-way ANOVA with a *post hoc* Bonferroni correction for multiple comparisons.

[3] N.A., not available.

[4] Three-way ANOVA was performed by R package of R, which didn't offer the sum of square after white adjustment (see Materials and Methods).

Table S8. Statistical test results for Figures 3B and S10.

Figure	Group	Time	*p*-Value (L-D)	*p*-Value (L-L)
Fig. 3B	Virgins vs. mated females	9:00	0.581	0.428
	(L-D)	10:00	0.422	0.658
	virgin (N = 6, n = 32)	11:00	0.637	0.847
	mated females (N = 8, n = 36)	12:00	0.372	0.15
	(L-L)	13:00	0.491	0.288
	virgin (N = 7, n = 29)	14:00	0.633	0.345
	mated females (N = 8, n = 33)	15:00	0.016	0.245
		16:00	0.014	0.32
		17:00	0.084	0.105
		18:00	0.253	0.535
		19:00	0.179	0.384
		20:00	0.105	0.173
		21:00	0.135	0.145
		22:00	0.003	0.002
		23:00	0.01	0.000324
		24:00	0.209	0.01
		1:00	0.049	0.003
		2:00	0.801	0.001
		3:00	0.534	0.000458
		4:00	0.598	0.009
		5:00	0.093	0.026
		6:00	0.978	0.412
		7:00	0.046	0.004
		8:00	0.025	0.014
Fig. S10A	Virgins vs. mated females	9:00	0.302	0.243
	(with VL2a innervations)	10:00	0.586	0.331
	(L-D)	11:00	0.582	0.454
	virgin (N = 5, n = 15)	12:00	0.508	0.97
	mated females (N = 8, n = 19)	13:00	0.092	0.495
	(L-L)	14:00	0.786	0.376
	virgin (N = 7, n = 14)	15:00	0.136	0.121
	mated females (N = 8, n = 19)	16:00	0.548	0.12
		17:00	0.15	0.056
		18:00	0.705	0.704
		19:00	0.694	0.944
		20:00	0.917	0.464
		21:00	0.782	0.501
		22:00	0.112	0.037
		23:00	0.246	0.114
		24:00	0.608	0.386
		1:00	0.641	0.296
		2:00	0.367	0.045
		3:00	0.898	0.083
		4:00	0.787	0.071
		5:00	0.229	0.093
		6:00	0.714	0.139
		7:00	0.115	0.031
		8:00	0.324	0.021

Fig. S10B	Virgins vs. mated females	9:00	0.19	0.368
	(No VL2a innervation)	10:00	0.171	0.104
	(L-D)	11:00	0.961	0.704
	virgin (N = 6, n = 17)	12:00	1	0.041
	mated females (N = 7, n = 17)	13:00	0.1	0.086
	(L-L)	14:00	0.812	0.269
	virgin (N = 6, n = 15)	15:00	0.37	0.855
	mated females (N = 5, n = 14)	16:00	0.323	0.15
		17:00	0.207	0.011
		18:00	0.944	0.64
		19:00	0.414	0.994
		20:00	0.813	0.968
		21:00	0.662	0.51
		22:00	0.834	0.649
		23:00	0.582	0.103
		24:00	0.339	0.365
		1:00	0.795	0.183
		2:00	0.693	0.337
		3:00	0.017	0.075
		4:00	0.879	0.079
		5:00	0.569	0.169
		6:00	0.409	0.946
		7:00	0.73	0.072
		8:00	0.7	0.733
Fig. S10C	No VL2a vs. with VL2a innervations	9:00	0.676	0.221
	(Mated females)	10:00	0.716	0.043
	(L-D)	11:00	0.991	0.457
	no VL2a (N = 7, n = 17)	12:00	0.296	0.282
	with VL2a (N = 8, n = 19)	13:00	0.000799	0.707
	(L-L)	14:00	0.043	0.256
	no VL2a (N = 5, n = 14)	15:00	0.678	0.264
	with VL2a (N = 8, n = 19)	16:00	0.083	0.443
		17:00	0.018	0.158
		18:00	0.914	0.608
		19:00	0.63	0.181
		20:00	0.78	0.397
		21:00	0.621	0.16
		22:00	0.388	0.088
		23:00	0.133	0.274
		24:00	0.381	0.816
		1:00	0.84	0.86
		2:00	0.992	0.108
		3:00	0.133	0.34
		4:00	0.568	0.545
		5:00	0.699	0.573
		6:00	0.428	0.164
		7:00	0.971	0.471
		8:00	0.518	0.068
Fig. S10D	No VL2a vs. with VL2a innervations	9:00	0.574	0.354
	(Virgins)	10:00	0.164	0.124

(L-D)	11:00	0.585	0.212
no VL2a (N = 6, n = 17)	12:00	0.782	0.212
with VL2a (N = 5, n = 15)	13:00	0.979	0.021
(L-L)	14:00	0.026	0.15
no VL2a (N = 6, n = 15)	15:00	0.669	0.768
with VL2a (N = 7, n = 14)	16:00	0.135	0.343
	17:00	0.501	0.007
	18:00	0.874	0.563
	19:00	0.476	0.091
	20:00	0.956	0.679
	21:00	0.545	0.024
	22:00	0.229	0.946
	23:00	0.675	0.475
	24:00	0.49	0.752
	1:00	0.604	0.51
	2:00	0.199	0.678
	3:00	0.78	0.319
	4:00	0.238	0.461
	5:00	0.175	0.982
	6:00	0.707	0.875
	7:00	0.135	0.987
	8:00	0.517	0.789

Three-way ANOVA was used to examine the main effects and interactions of variables and the *post hoc* Bonferroni correction was used to analyze multiple comparisons among different conditions. *, $p < 0.05$; **, $p < 0.01$; ***, $p < 0.001$.

Table S9. Genotypes of flies used in experiments described in Figures 1-4 and S1-S13.

Figure	Genotype
**Fig. 1A (left)**	*y[1] w[*];UAS-nuclacZ UAS-mCD8GFP/+;OK107-GAL4/+*
**Fig. 1A (right)**	*y[1] w[*]/w[*];GAD1-GAL80 (#11-2)/UAS-nuclacZ UAS-mCD8GFP;;OK107-GAL4/+*
**Fig. 1B, 1D, 1F**	*y[1] w[*];GAD1-GAL80 (#11-2)/UAS-nuclacZ UAS-mCD8GFP;;OK107-GAL4/+*<br>(or) *y[1] w[*]/Y;GAD1-GAL80 (#11-2)/UAS-nuclacZ UAS-mCD8GFP;;OK107-GAL4/+*
**Fig. 1E (top)**	*y[1] w[*]/Y;GAD1-GAL80 (#11-2)/UAS-nuclacZ UAS-mCD8GFP;;OK107-GAL4/+*
**Fig. 1E (bottom)**	*y[1] w[*];GAD1-GAL80 (#11-2)/UAS-nuclacZ UAS-mCD8GFP ;OK107-GAL4/+*
**Fig. 2A (top)**	*y[1] w[*]/Y;GAD1-GAL80 (#11-2)/UAS-nuclacZ UAS-mCD8GFP; OK107-GAL4/+*
**Fig. 2A (bottom)**	*y[1] w[*];GAD1-GAL80 (#11-2)/UAS-nuclacZ UAS-mCD8GFP;;OK107-GAL4/+*
**Fig. 2B (left)**	*y[1] w[*];GAD1-GAL80 (#11-2)/UAS-nuclacZ UAS-mCD8GFP;;OK107-GAL4/+*<br>(or) *y[1] w[*]/Y;GAD1-GAL80 (#11-2)/UAS-nuclacZ UAS-mCD8GFP;;OK107-GAL4/+*
**Fig. 2B (right)**	*w[1118];GAD1-GAL80 (#11-2)/UAS-mCD8GFP.1;;OK107-GAL4/+* (*Canton S* background)<br>(or) *w[1118]/Y GAD1-GAL80 (#11-2)/UAS-mCD8GFP.1;;OK107-GAL4/+* (*Canton S* background)
**Fig. 2C (top)**	*y[1] w[*]/Y;GAD1-GAL80 (#11-2)/UAS-nuclacZ UAS-mCD8GFP; OK107-GAL4/+*
**Fig. 2C (bottom)**	*y[1] w[*];GAD1-GAL80 (#11-2)/UAS-nuclacZ UAS-mCD8GFP;;OK107-GAL4/+*
**Fig. 2D**	*y[1] w[*];GAD1-GAL80 (#11-2)/UAS-nuclacZ UAS-mCD8GFP;;OK107-GAL4/+*
**Fig. 2E**	*y[1] w[*]/Y;GAD1-GAL80 (#11-2)/UAS-nuclacZ UAS-mCD8GFP;;OK107-GAL4/+*
**Fig. 2F (left)**	*y[1] w[*]/Y;UAS-FRT-stop-FRT-mCD8GFP/+;fru-FLP[BD]/+;OK107/+*
**Fig. 2F (right)**	*y[1] w[*]/w[*];UAS-FRT-stop-FRT-mCD8GFP/+;fru-FLP[BD]/+;OK107/+*
**Fig. 2G (left)**	*w[*]/Y;UAS-FLP/13xlexAop2(FRT.stop)myr::smGdP-V5 (attP 40);dsx-GAL4/tubP-GAL80[ts] (2), nSyb-LexA.DBD::QF.AD (attP 2);*
**Fig. 2G (right)**	*w[*]/w[*];UAS-FLP/13xlexAop2(FRT.stop)myr::smGdP-V5 (attP 40);dsx-GAL4/tubP-GAL80[ts] (2), nSyb-LexA.DBD::QF.AD (attP 2);*
**Fig. 3B, 3C**	*w[1118];GAD1-GAL80 (#11-2)/UAS-mCD8GFP;;OK107-GAL4/+* (*Canton S* background)
**(males for mating, Fig. 3B, 3C)**	*w[1118]/Y;GAD1-GAL80 (#11-2)/UAS-mCD8GFP;;OK107-GAL4/+* (*Canton S* background)
**Fig. 4D (left), 4E**	*w[*] Peb-QF2/y[1] w[*];QUAS-CD4-spGFP11 (#22-2)/GAD1-GAL80 (#11-2);UAS-CD4-spGFP1-10/UAS-CD4-spGFP1-10;OK107-GAL4/+*
**Fig. 4D (middle), 4E**	*w[*]/y[1] w[*];GH146-QF (#11) QUAS-CD4-spGFP11 (#22-2)/GAD1-GAL80 (#11-2);UAS-CD4-spGFP1-10/UAS-CD4-spGFP1-10;OK107-GAL4/+*
**Fig. 4D (right), 4E**	*w[*]/y[1] w[*];QUAS-CD4-spGFP11 (#22-2)/GAD1-GAL80 (#11-2);449-QF (#1-3) UAS-CD4-spGFP1-10/UAS-CD4-spGFP1-10;OK107-GAL4/+*
**Fig. 4F (top, middle, bottom)**	*y[1] w[*] P{UAS-myrGFP.QUAS-mtdTomato-3xHA} attP8/y[1] w[*];P{trans-Tango} attP40/GAD1GAL80 (#11-2);;OK107-GAL4/+*
**Fig. 4G**	*y[1] w[*] P{UAS-myrGFP.QUAS-mtdTomato-3xHA} attP8/y[1] w[*];P{trans-Tango} attP40/GAD1GAL80 (#11-2);;OK107-GAL4/+*
**fig. S1A, S1B**	*y[1] w[*];GAD1-GAL80 (#11-2)/UAS-nuclacZ UAS-mCD8GFP;;OK107-GAL4/+*
	(or) *y[1] w[*]/Y;GAD1-GAL80 (#11-2)/UAS-nuclacZ UAS-mCD8GFP;;OK107-GAL4/+*
**fig. S1C, S1I**	*y[1] w[*];GAD1-GAL80 (#11-2)/UAS-nuclacZ UAS-mCD8GFP;;OK107-GAL4/+*
**fig. S1D, S1E, S1F, S1G, S1H**	*y[1] w[*]/Y;GAD1-GAL80 (#11-2)/UAS-nuclacZ UAS-mCD8GFP;;OK107-GAL4/+*

**fig. S3A**	*y[1] w[*];GAD1-GAL80 (#11-2)/UAS-nuclacZ UAS-mCD8GFP ;OK107-GAL4/+*<br>(or) *y[1] w[*]/Y;GAD1-GAL80 (#11-2)/UAS-nuclacZ UAS-mCD8GFP;;OK107-GAL4/+*<br>(or) *w[1118];GAD1-GAL80 (#11-2)/UAS-mCD8GFP;;OK107-GAL4/+* (*Canton S* background)<br>(or) *w[1118]/Y;GAD1-GAL80 (#11-2)/UAS-mCD8GFP;;OK107-GAL4/+* (*Canton S* background)
**fig. S3B, S3C**	*y[1] w[*];GAD1-GAL80 (#11-2)/UAS-nuclacZ UAS-mCD8GFP; ;OK107-GAL4/+*<br>(or) *y[1] w[*]/Y;GAD1-GAL80 (#11-2)/UAS-nuclacZ UAS-mCD8GFP; OK107-GAL4/+*
**fig. S3D (top), S3E (top)**	*y[1] w[*]/Y;GAD1-GAL80 (#11-2)/UAS-nuclacZ UAS-mCD8GFP;;OK107-GAL4/+*
**fig. S3D (bottom), S3E (bottom)**	*y[1] w[*];GAD1-GAL80 (#11-2)/UAS-nuclacZ UAS-mCD8GFP;;OK107-GAL4/+*
**fig. S3F**	*w[1118];GAD1-GAL80 (#11-2)/UAS-mCD8GFP;;OK107-GAL4/+* (*Canton S* background)<br>(or) *w[1118]/Y;GAD1-GAL80 (#11-2)/UAS-mCD8GFP;;OK107-GAL4/+* (*Canton S* background)
**fig. S3G (top)**	*w[1118]/Y;GAD1-GAL80 (#11-2)/UAS-mCD8GFP;; OK107-GAL4/+* (*Canton S* background)
**fig. S3G (bottom)**	*w[1118];GAD1-GAL80 (#11-2)/UAS-mCD8GFP;;OK107-GAL4/+* (*Canton S* background)
**fig. S3H**	*w[*]/w[*];GAD1-GAL80 (#11-2)/UAS-mCD8GFP;fru[sat]/+;OK107-GAL4/+*<br>(or) *w[*]/w[*];GAD1-GAL80 (#11-2)/UAS-mCD8GFP fru[2]/+;OK107-GAL4/+*<br>(or) *w[*]/w[*];GAD1-GAL80 (#11-2)/UAS-mCD8GFP;fru[sat]/fru[2];OK107-GAL4/+*<br>(or) *w[*] SPR[attp]/w[*];GAD1-GAL80 (#11-2)UAS-mCD8GFP.1/+ ;OK107-GAL4/+*<br>(or) *w[*] SPR[attp]/w[*] SPR[attp]; GAD1-GAL80 (#11-2) UAS-mCD8GFP.1/+; ;OK107-GAL4/+*<br>(or) *w[*]/w[*];GAD1-GAL80 (#11-2)/UAS-nuclacZ UAS-mCD8GFP; Mip[attp]/+;OK107-GAL4/+*<br>(or) *w[*]/w[*];GAD1-GAL80 (#11-2)/UAS-nuclacZ UAS-mCD8GFP;Mip[attp]/Mip[attp]; OK107-GAL4/+*
**fig. S4**	*y[1] w[*];GAD1-GAL80 (#11-2)/UAS-nuclacZ UAS-mCD8GFP; ;OK107-GAL4/+*<br>(or) *y[1] w[*]/Y GAD1-GAL80 (#11-2)/UAS-nuclacZ UAS-mCD8GFP;; OK107-GAL4/+*
**fig. S5A (top), S5B (top)**	*y[1] w[*];GAD1-GAL80 (#11-2)/UAS-nuclacZ UAS-mCD8GFP;;OK107-GAL4/+*<br>(or) *y[1] w[*]/Y;GAD1-GAL80 (#11-2)/UAS-nuclacZ UAS-mCD8GFP;;OK107-GAL4/+*
**fig. S5A (middle), S5B (middle)**	*y[1] w[*]/Y;GAD1-GAL80 (#11-2)/UAS-nuclacZ UAS-mCD8GFP OK107-GAL4/+*
**fig. S5A (bottom), S5B (bottom)**	*y[1] w[*];GAD1-GAL80 (#11-2)/UAS-nuclacZ UAS-mCD8GFP;;OK107-GAL4/+*
**fig. S5C**	*y[1] w[*];GAD1-GAL80 (#11-2)/UAS-nuclacZ UAS-mCD8GFP;;OK107-GAL4/+*<br>(or) *y[1] w[*]/Y;GAD1-GAL80 (#11-2)/UAS-nuclacZ UAS-mCD8GFP;;OK107-GAL4/+*
**fig. S5D (left)**	*y[1] w[*]; GAD1-GAL80 (#11-2)/UAS-nuclacZ UAS-mCD8GFP; ; OK107-GAL4/+*<br>(or) *y[1] w[*]/Y;GAD1-GAL80 (#11-2)/UAS-nuclacZ UAS-mCD8GFP;;OK107-GAL4/+*
**fig. S5D (right)**	*w[1118];GAD1-GAL80 (#11-2)/UAS-mCD8GFP.1;;OK107-GAL4/+* (*Canton S* background)<br>(or) *w[1118]/Y;GAD1-GAL80 (#11-2)/UAS-mCD8GFP.1;;OK107-GAL4/+* (*Canton S* background)
**fig. S5E**	*y[1] w[*];GAD1-GAL80 (#11-2)/UAS-nuclacZ UAS-mCD8GFP;;OK107-GAL4/+*<br>(or) *y[1] w[*]/Y;GAD1-GAL80 (#11-2)/UAS-nuclacZ UAS-mCD8GFP;;OK107-GAL4/+*
**fig. S6A, S5B**	*y[1] w[*];GAD1-GAL80 (#11-2)/UAS-nuclacZ UAS-mCD8GFP;;OK107-GAL4/+*<br>(or) *y[1] w[*]/Y GAD1-GAL80 (#11-2)/UAS-nuclacZ UAS-mCD8GFP;;OK107-GAL4/+*

**fig. S6C (left)**	*y[1] w[*];GAD1-GAL80 (#11-2)/UAS-nuclacZ UAS-mCD8GFP;;OK107-GAL4/+*<br>(or) *y[1] w[*]/Y;GAD1-GAL80 (#11-2)/UAS-nuclacZ UAS-mCD8GFP;;OK107-GAL4/+*
**fig. S6C (right-left)**	*y[1] w[*]/Y;GAD1-GAL80 (#11-2)/UAS-nuclacZ UAS-mCD8GFP;;OK107-GAL4/+*
**fig. S6C (right-right)**	*y[1] w[*]; GAD1-GAL80 (#11-2)/UAS-nuclacZ UAS-mCD8GFP;;OK107-GAL4/+*
**fig. S7A**	*y[1] w[*]/Y;GAD1-GAL80 (#11-2)/UAS-nuclacZ UAS-mCD8GFP; ; OK107-GAL4/+*
**fig. S7B (left)**	*y[1] w[*]/Y;GAD1-GAL80 (#11-2)/UAS-nuclacZ UAS-mCD8GFP;fru-GAL4[BD]/+;*
**fig. S7B (right)**	*y[1] w[*]/y[1] w[*];GAD1-GAL80 (#11-2)/ UAS-nuclacZ UAS-mCD8GFP fru-GAL4[BD]/+;*
**fFig. S7C (top-left)**	*y[1] w[*]/Y;UAS-FRT-stop-FRT-mCD8GFP/+ fru-FLP[BD]/+;OK107/+*
**fig. S7C (top-right)**	*y[1] w[*]/w[*];UAS-FRT-stop-FRT-mCD8GFP/+; fru-FLP[BD]/+;OK107/+*
**fig. S7C (middle-left)**	*y[1] w[*]/Y;UAS-FRT-stop-FRT-mCD8GFP/OK371-GAL4;fru-FLP[BD]/+;*
**fig. S7C (middle-right)**	*y[1] w[*]/w[1118];UAS-FRT-stop-FRT-mCD8GFP/OK371-GAL4;fru-FLP[BD]/+;*
**fig. S7C (bottom-left)**	*y[1] w[*]/Y;UAS-FRT-stop-FRT-mCD8GFP/+;fru-FLP[BD]/DvGlut[CNSIII]-GAL4;*
**fig. S7C (bottom-right)**	*y[1] w[*]/w[*];UAS-FRT-stop-FRT-mCD8GFP/+;fru-FLP[BD]/DvGlut[CNSIII]-GAL4;*
**fig. S7d**	(Control $fru^{+/-}$) *w[*]/w[*];GAD1-GAL80 (#11-2)/UAS-mCD8GFP.1;fru[sat]/+;OK107-GAL4/+*<br>(or) *w[*]/w[*];GAD1-GAL80 (#11-2)/UAS-mCD8GFP.1;fru[2]/+;OK107-GAL4/+*
**fig. S7d**	(Mutant $fru^{-/-}$) *w[*]/w[*];GAD1-GAL80 (#11-2)/UAS-mCD8GFP.1 fru[sat]/ fru[2];OK107-GAL4/+*
**(males for mating, fig. S7D)**	*y w/Y; GAD1-GAL80 (#11-2)/UAS-nuclacZ UAS-mCD8GFP;;OK107-GAL4/+*
**fig. S7E (left)**	*w[1118]/Y;PBac{y[+mDint2] w[+mC]=dsx-GFP.FPTB}VK00037*
**fig. S7E (right)**	*w[1118];PBac{y[+mDint2] w[+mC]=dsx-GFP.FPTB}VK00037*
**fig. S7F (left)**	*w[*]/Y;UAS-FLP/13xlexAop2(FRT.stop)myr::smGdP-V5 (attP 40);dsx-GAL4/tubP-GAL80[ts] (2), nSyb-LexA.DBD::QF.AD (attP 2)*
**fig. S7F (right)**	*w[*]/w[*];UAS-FLP/13xlexAop2(FRT.stop)myr::smGdP-V5 (attP 40);dsx-GAL4/tubP-GAL80[ts] (2), nSyb-LexA.DBD::QF.AD (attP 2)*
**fig. S8A, S8B, S8C**	*y w;GAD1-GAL80 (#11-2)/UAS-nuclacZ UAS-mCD8GFP;;OK107-GAL4/+*
**(males for mating, fig. S8A)**	(control) *+/Y;;P{PZ}bol[1] ry[506]/TM6B Tb[1]*<br>(*bol* mutants, fertile or sterile) *+/Y;P{PZ}bol[1] ry[506]/ P{PZ}bol[1] ry[506];*
**(males for mating, fig. S8B)**	(control) *w[*]/Y;;SP[CTL]/SP[CTL];*<br>(*SP* mutant) *w[*]/Y;;SP[0325]/SP[0325];*
**(males for mating, fig. S8C)**	(control) *+/Y;UAS-Stinger II/+; PromE(800)-GAL4[4M] tubGAL80[ts]/+;*<br>(oenocyte-less) *+/Y;UAS-hid UAS-Stinger II/+;PromE(800)-GAL4[4M] tubGAL80[ts]/+;*
**fig. S9B, S9C**	*Canton S*
**fig. S9D (top)**	*Canton S*
**fig. S9D (bottom)**	*Oregon R*
**fig. S10A, S10B, S10C, S10D**	*w[1118] GAD1-GAL80 (#11-2)/UAS-mCD8GFP;;OK107-GAL4/+* (*Canton S* background)
**(males for mating, fig. S10A, S10B, S10C, S10D)**	*w[1118]/Y;GAD1-GAL80 (#11-2)/UAS-mCD8GFP;;OK107-GAL4/+* (*Canton S* background)
**fig. S11A**	*y[1] w[*] GAD1-GAL80 (#11-2)/UAS-nuclacZ UAS-mCD8GFP;;OK107-GAL4/+*
**fig. S11B**	*y[1] w[*];GAD1-GAL80 (#11-2)/UAS-nuclacZ UAS-mCD8GFP;;OK107-GAL4/+*

**fig. S11C**	*y[1] w[*]/w[*];GAD1-GAL80 (#11-2)/UAS-nuclacZ UAS-mCD8GFP; UAS-syt-HA/+;OK107-GAL4/+*
**fig. S13B (left), S13C, S13D**	*w[*] Peb-QF2/y[1] w[*];QUAS-CD4GFP11 (#22-2)/GAD1-GAL80 (#11-2);UAS-CD4GFP1-10/UAS-CD4GFP1-10;OK107-GAL4/+*
**fig. S13B (middle), S13C, S13D**	*w[*]/y[1] w[*];GH146-QF (#11) QUAS-CD4GFP11 (#22-2)/GAD1-GAL80 (#11-2);UAS-CD4GFP1-10/UAS-CD4GFP1-10;OK107-GAL4/+*
**fig. S13B (right), S13C, S14D**	*w[*]/y[1] w[*];QUAS-CD4GFP11 (#22-2)/GAD1-GAL80 (#11-2) 449-QF (#1-3) UAS-CD4GFP1-10/UAS-CD4GFP1-10;OK107-GAL4/+*
**fig. S13F, S13G**	*y[1] w[*] P{UAS-myrGFP.QUAS-mtdTomato-3xHA} attP8/y[1] w[*];P{trans-Tango} attP40/GAD1GAL80 (#11-2);;OK107-GAL4/+*

REFERENCES AND NOTES

1. D. J. Schulz, J. M. Goaillard, E. Marder, Variable channel expression in identified single and electrically coupled neurons in different animals. *Nat. Neurosci.* **9**, 356–362 (2006).

2. M. Langen, E. Agi, D. J. Altschuler, L. F. Wu, S. J. Altschuler, P. R. Hiesinger, The developmental rules of neural superposition in *Drosophila*. *Cell* **162**, 120–133 (2015).

3. G. A. Linneweber, M. Andriatsilavo, S. B. Dutta, M. Bengochea, L. Hellbruegge, G. Liu, R. K. Ejsmont, A. D. Straw, M. Wernet, P. R. Hiesinger, B. A. Hassan, A neurodevelopmental origin of behavioral individuality in the *Drosophila* visual system. *Science* **367**, 1112–1119 (2020).

4. S. J. Caron, V. Ruta, L. F. Abbott, R. Axel, Random convergence of olfactory inputs in the *Drosophila* mushroom body. *Nature* **497**, 113–117 (2013).

5. Y. H. Chou, M. L. Spletter, E. Yaksi, J. C. S. Leong, R. I. Wilson, L. Luo, Diversity and wiring variability of olfactory local interneurons in the *Drosophila* antennal lobe. *Nat. Neurosci.* **13**, 439–449 (2010).

6. B. Wamsley, G. Fishell, Genetic and activity-dependent mechanisms underlying interneuron diversity. *Nat. Rev. Neurosci.* **18**, 299–309 (2017).

7. N. Randel, R. Shahidi, C. Verasztó, L. A. Bezares-Calderón, S. Schmidt, G. Jékely, Inter-individual stereotypy of the Platynereis larval visual connectome. *eLife* **4**, e08069 (2015).

8. J. Lu, J. C. Tapia, O. L. White, J. W. Lichtman, The interscutularis muscle connectome. *PLOS Biol.* **7**, e1000032 (2009).

9. J. Winnubst, E. Bas, T. A. Ferreira, Z. Wu, M. N. Economo, P. Edson, B. J. Arthur, C. Bruns, K. Rokicki, D. Schauder, D. J. Olbris, S. D. Murphy, D. G. Ackerman, C. Arshadi, P. Baldwin, R. Blake, A. Elsayed, M. Hasan, D. Ramirez, B. D. Santos, M. Weldon, A. Zafar, J. T. Dudman, C. R. Gerfen, A. W. Hantman, W. Korff, S. M. Sternson, N. Spruston, K. Svoboda, J. Chandrashekar, Reconstruction of 1,000 projection neurons reveals new cell types and organization of long-range connectivity in the mouse brain. *Cell* **179**, 268–281.e13 (2019).

10. R. J. Johnston Jr, C. Desplan, Interchromosomal communication coordinates intrinsically stochastic expression between alleles. *Science* **343**, 661–665 (2014).

11. T. Hige, Y. Aso, M. N. Modi, G. M. Rubin, G. C. Turner, Heterosynaptic plasticity underlies aversive olfactory learning in *Drosophila*. *Neuron* **88**, 985–998 (2015).

12. K. S. Honegger, M. A. Smith, M. A. Churgin, G. C. Turner, B. L. de Bivort, Idiosyncratic neural coding and neuromodulation of olfactory individuality in *Drosophila*. *Proc. Natl. Acad. Sci. U.S.A.* **117**, 23292–23297 (2020).

13. C. S. Goodman, Isogenic grasshoppers: Genetic variability in the morphology of identified neurons. *J. Comp. Neurol.* **182**, 681–705 (1978).

14. S. J. Zottoli, Comparison of Mauthner cell size in teleosts. *J. Comp. Neurol.* **178**, 741–757 (1978).

15. L. B. Vosshall, R. F. Stocker, Molecular architecture of smell and taste in *Drosophila*. *Annu. Rev. Neurosci.* **30**, 505–533 (2007).

16. R. I. Wilson, Early olfactory processing in Drosophila: Mechanisms and principles. *Annu. Rev. Neurosci.* **36**, 217–241 (2013).

17. P. Schlegel, A. S. Bates, T. Stürner, S. R. Jagannathan, N. Drummond, J. Hsu, L. Serratosa Capdevila, A. Javier, E. C. Marin, A. Barth-Maron, I. F.M. Tamimi, F. Li, G. M. Rubin, S. M. Plaza, M. Costa, G. S. X. E. Jefferis, Information flow, cell types and stereotypy in a full olfactory connectome. *eLife* **10**, e66018 (2021).

18. L. K. Scheffer, C. S. Xu, M. Januszewski, Z. Lu, S.Y. Takemura, K. J. Hayworth, G. B. Huang, K. Shinomiya, J. Maitlin-Shepard, S. Berg, J. Clements, P. M. Hubbard, W. T. Katz, L. Umayam, T. Zhao, D. Ackerman, T. Blakely, J. Bogovic, T. Dolafi, D. Kainmueller, T. Kawase, K. A. Khairy, L. Leavitt, P. H. Li, L. Lindsey, N. Neubarth, D. J. Olbris, H. Otsuna, E. T. Trautman, M. Ito, A. S. Bates, J. Goldammer, T. Wolff, R. Svirskas, P. Schlegel, E. Neace, C. J. Knecht, C. X. Alvarado, D. A. Bailey, S. Ballinger, J. A. Borycz, B. S. Canino, N. Cheatham, M. Cook, M. Dreher, O. Duclos, B. Eubanks, K. Fairbanks, S. Finley, N. Forknall, A. Francis, G. P. Hopkins, E. M. Joyce, S.J. Kim, N. A. Kirk, J. Kovalyak, S. A. Lauchie, A. Lohff, C. Maldonado, E. A. Manley, S. McLin, C.

Mooney, M. Ndama, O. Ogundeyi, N. Okeoma, C. Ordish, N. Padilla, C. M. Patrick, T. Paterson, E. E. Phillips, E. M. Phillips, N. Rampally, C. Ribeiro, M. K. Robertson, J. T. Rymer, S. M. Ryan, M. Sammons, A. K. Scott, A. L. Scott, A. Shinomiya, C. Smith, K. Smith, N. L. Smith, M. A. Sobeski, A. Suleiman, J. Swift, S. Takemura, I. Talebi, D. Tarnogorska, E. Tenshaw, T. Tokhi, J. J. Walsh, T. Yang, J. A. Horne, F. Li, R. Parekh, P. K. Rivlin, V. Jayaraman, M. Costa, G. S.X.E. Jefferis, K. Ito, S. Saalfeld, R. George, I. A. Meinertzhagen, G. M. Rubin, H. F. Hess, V. Jain, S. M. Plaza, A connectome and analysis of the adult Drosophila central brain. *eLife* **9**, e57443 (2020).

19. Z. Zheng, J. S. Lauritzen, E. Perlman, C. G. Robinson, M. Nichols, D. Milkie, O. Torrens, J. Price, C. B. Fisher, N. Sharifi, S. A. Calle-Schuler, L. Kmecova, I. J. Ali, B. Karsh, E. T. Trautman, J. A. Bogovic, P. Hanslovsky, Gregory S X E Jefferis, M. Kazhdan, K. Khairy, S. Saalfeld, R. D. Fetter, D. D. Bock, A complete electron microscopy volume of the brain of adult *Drosophila melanogaster*. *Cell* **174**, 730–743.e22 (2018).

20. A. Couto, M. Alenius, B. J. Dickson, Molecular, anatomical, and functional organization of the Drosophila olfactory system. *Curr. Biol.* **15**, 1535–1547 (2005).

21. E. Fishilevich, L. B. Vosshall, Genetic and functional subdivision of the *Drosophila* antennal lobe. *Curr. Biol.* **15**, 1548–1553 (2005).

22. P. Stockinger, D. Kvitsiani, S. Rotkopf, L. Tirian, B. J. Dickson, Neural circuitry that governs *Drosophila* male courtship behavior. *Cell* **121**, 795–807 (2005).

23. G. S. Jefferis, C. J. Potter, A. M. Chan, E. C. Marin, T. Rohlfing, C. R. Maurer Jr, L. Luo, Comprehensive maps of *Drosophila* higher olfactory centers: Spatially segregated fruit and pheromone representation. *Cell* **128**, 1187–1203 (2007).

24. Y. Grosjean, R. Rytz, J.P. Farine, L. Abuin, J. Cortot, G. S. X. E. Jefferis, R. Benton, An olfactory receptor for food-derived odours promotes male courtship in *Drosophila*. *Nature* **478**, 236–240 (2011).

25. K. Kimura, M. Ote, T. Tazawa, D. Yamamoto, Fruitless specifies sexually dimorphic neural circuitry in the *Drosophila* brain. *Nature* **438**, 229–233 (2005).

26. D. S. Manoli, M. Foss, A. Villella, B. J. Taylor, J. C. Hall, B. S. Baker, Male-specific fruitless specifies the neural substrates of *Drosophila* courtship behaviour. *Nature* **436**, 395–400 (2005).

27. E. J. Rideout, A. J. Dornan, M. C. Neville, S. Eadie, S. F. Goodwin, Control of sexual differentiation and behavior by the doublesex gene in *Drosophila melanogaster*. *Nat. Neurosci.* **13**, 458–466 (2010).

28. K. Kimura, T. Hachiya, M. Koganezawa, T. Tazawa, D. Yamamoto, Fruitless and doublesex coordinate to generate male-specific neurons that can initiate courtship. *Neuron* **59**, 759–769 (2008).

29. S. Cachero, A. D. Ostrovsky, J. Y. Yu, B. J. Dickson, G. S. Jefferis, Sexual dimorphism in the fly brain. *Curr. Biol.* **20**, 1589–1601 (2010).

30. J. Y. Yu, M. I. Kanai, E. Demir, G. S. Jefferis, B. J. Dickson, Cellular organization of the neural circuit that drives *Drosophila* courtship behavior. *Curr. Biol.* **20**, 1602–1614 (2010).

31. H. Liu, E. Kubli, Sex-peptide is the molecular basis of the sperm effect in *Drosophila melanogaster*. *Proc. Natl. Acad. Sci. U.S.A.* **100**, 9929–9933 (2003).

32. S. Zawistowski, R. C. Richmond, Inhibition of courtship and mating of *Drosophila melanogaster* by the male-produced lipid, cis-vaccenyl acetate. *J. Insect Physiol.* **32**, 189–192 (1986).

33. C. Everaerts, J. P. Farine, M. Cobb, J. F. Ferveur, *Drosophila* cuticular hydrocarbons revisited: mating status alters cuticular profiles. *PLOS ONE* **5**, e9607 (2010).

34. E. D. Hoopfer, A. Penton, R. J. Watts, L. Luo, Genomic analysis of *Drosophila* neuronal remodeling: a role for the RNA-binding protein Boule as a negative regulator of axon pruning. *J. Neurosci.* **28**, 6092–6103 (2008).

35. J. C. Billeter, J. Atallah, J. J. Krupp, J. G. Millar, J. D. Levine, Specialized cells tag sexual and species identity in *Drosophila melanogaster*. *Nature* **461**, 987–991 (2009).

36. J. Peng, S. Chen, S. Büsser, H. Liu, T. Honegger, E. Kubli, Gradual release of sperm bound sex-peptide controls female postmating behavior in *Drosophila*. *Curr. Biol.* **15**, 207–213 (2005).

37. G. B. Carvalho, P. Kapahi, D. J. Anderson, S. Benzer, Allocrine modulation of feeding behavior by the sex peptide of Drosophila. *Curr. Biol.* **16**, 692–696 (2006).

38. J. Ro, Z. M. Harvanek, S. D. Pletcher, FLIC: High-throughput, continuous analysis of feeding behaviors in Drosophila. *PLOS ONE* **9**, e101107 (2014).

39. M. P. Fernandez, J. Berni, M. F. Ceriani, Circadian remodeling of neuronal circuits involved in rhythmic behavior. *PLOS Biol.* **6**, e69 (2008).

40. W. W. Liu, R. I. Wilson, Glutamate is an inhibitory neurotransmitter in the Drosophila olfactory system. *Proc. Natl. Acad. Sci. U.S.A.* **110**, 10294–10299 (2013).

41. E. H. Feinberg, M. K. VanHoven, A. Bendesky, G. Wang, R. D. Fetter, K. Shen, C. I. Bargmann, GFP reconstitution across synaptic partners (GRASP) defines cell contacts and synapses in living nervous systems. *Neuron* **57**, 353–363 (2008).

42. N. F. Liou, S.H. Lin, Y.J. Chen, K.T. Tsai, C.J. Yang, T.Y. Lin, T.H. Wu, H.J. Lin, Y.T. Chen, D. M. Gohl, M. Silies, Y.H. Chou, Diverse populations of local interneurons integrate into the Drosophila adult olfactory circuit. *Nat. Commun.* **9**, 2232 (2018).

43. M. Talay, E. B. Richman, N. J. Snell, G. G. Hartmann, J. D. Fisher, A. Sorkaç, J. F. Santoyo, C. Chou-Freed, N. Nair, M. Johnson, J. R. Szymanski, G. Barnea, Transsynaptic mapping of second-order taste neurons in flies by trans-Tango. *Neuron* **96**, 783–795.e4 (2017).

44. D. Berdnik, T. Chihara, A. Couto, L. Luo, Wiring stability of the adult Drosophila olfactory circuit after lesion. *J. Neurosci.* **26**, 3367–3376 (2006).

45. J. M. Devaud, A. Acebes, A. Ferrus, Odor exposure causes central adaptation and morphological changes in selected olfactory glomeruli in Drosophila. *J. Neurosci.* **21**, 6274–6282 (2001).

46. S. Sachse, E. Rueckert, A. Keller, R. Okada, N. K. Tanaka, K. Ito, L. B. Vosshall, Activity-dependent plasticity in an olfactory circuit. *Neuron* **56**, 838–850 (2007).

47. S. Das, M. K. Sadanandappa, A. Dervan, A. Larkin, J. A. Lee, I. P. Sudhakaran, R. Priya, R. Heidari, E. E. Holohan, A. Pimentel, A. Gandhi, K. Ito, S. Sanyal, J. W. Wang, V. Rodrigues, M. Ramaswami, Plasticity of local GABAergic interneurons drives olfactory habituation. *Proc. Natl. Acad. Sci. U.S.A.* **108**, E646–E654 (2011).

48. T. Lee, L. Luo, Mosaic analysis with a repressible cell marker for studies of gene function in neuronal morphogenesis. *Neuron* **22**, 451–461 (1999).

49. C. J. Potter, B. Tasic, E. V. Russler, L. Liang, L. Luo, The Q system: A repressible binary system for transgene expression, lineage tracing, and mosaic analysis. *Cell* **141**, 536–548 (2010).

50. M. D. Gordon, K. Scott, Motor control in a Drosophila taste circuit. *Neuron* **61**, 373–384 (2009).

51. B. D. Pfeiffer, T.T. B. Ngo, K. L. Hibbard, C. Murphy, A. Jenett, J. W. Truman, G. M. Rubin, Refinement of tools for targeted gene expression in Drosophila. *Genetics* **186**, 735–755 (2010).

52. S. E. McGuire, P. T. Le, A. J. Osborn, K. Matsumoto, R. L. Davis, Spatiotemporal rescue of memory dysfunction in Drosophila. *Science* **302**, 1765–1768 (2003).

53. S. Jagadish, G. Barnea, T. R. Clandinin, R. Axel, Identifying functional connections of the inner photoreceptors in Drosophila using Tango-Trace. *Neuron* **83**, 630–644 (2014).

54. R. W. Daniels, M. V. Gelfand, C. A. Collins, A. DiAntonio, Visualizing glutamatergic cell bodies and synapses in Drosophila larval and adult CNS. *J. Comp. Neurol.* **508**, 131–152 (2008).

55. C. C. Lin, C. J. Potter, Editing transgenic DNA components by inducible gene replacement in *Drosophila melanogaster*. *Genetics* **203**, 1613–1628 (2016).

56. Y. J. Chen, H.H. Chang, S.H. Lin, T.Y. Lin, T.H. Wu, H.J. Lin, N.F. Liou, C.J. Yang, Y.T. Chen, K. H. Chang, C.Y. Li, Y.H. Chou, Differential efficacy of genetically swapping GAL4. *J. Neurogenet.* **33**, 52–63 (2019).

57. H. Katow, T. Takahashi, K. Saito, H. Tanimoto, S. Kondo, Tango knock-ins visualize endogenous activity of G protein-coupled receptors in Drosophila. *J. Neurogenet.* **33**, 44–51 (2019).

58. M. M. Kudron, A. Victorsen, L. Gevirtzman, L.D. W. Hillier, W. W. Fisher, D. Vafeados, M. Kirkey, A. S. Hammonds, J. Gersch, H. Ammouri, M. L. Wall, J. Moran, D. Steffen, M. Szynkarek, S. Seabrook-Sturgis, N. Jameel, M. Kadaba, J. Patton, R. Terrell, M. Corson, T. J. Durham, S. Park, S. Samanta, M. Han, J. Xu, K.K. Yan, S. E. Celniker, K. P. White, L. Ma, M. Gerstein, V. Reinke, R. H. Waterston, The ModERN resource: Genome-wide binding profiles for hundreds of *Drosophila* and *Caenorhabditis elegans* transcription factors. *Genetics* **208**, 937–949 (2018).

59. H. Ito, K. Sato, M. Koganezawa, M. Ote, K. Matsumoto, C. Hama, D. Yamamoto, Fruitless recruits two antagonistic chromatin factors to establish single-neuron sexual dimorphism. *Cell* **149**, 1327–1338 (2012).

60. B. Deng, Q. Li, X. Liu, Y. Cao, B. Li, Y. Qian, R. Xu, R. Mao, E. Zhou, W. Zhang, J. Huang, Y. Rao, Chemoconnectomics: Mapping chemical transmission in *Drosophila*. *Neuron* **101**, 876–893.e4 (2019).

61. M. Ng, R. D. Roorda, S. Q. Lima, B. V. Zemelman, P. Morcillo, G. Miesenböck, Transmission of olfactory information between three populations of neurons in the antennal lobe of the fly. *Neuron* **36**, 463–474 (2002).

62. A. I. Saeed, V. Sharov, J. White, J. Li, W. Liang, N. Bhagabati, J. Braisted, M. Klapa, T. Currier, M. Thiagarajan, A. Sturn, M. Snuffin, A. Rezantsev, D. Popov, A. Ryltsov, E. Kostukovich, I. Borisovsky, Z. Liu, A. Vinsavich, V. Trush, J. Quackenbush, TM4: A free, open-source system for microarray data management and analysis. *Biotechniques* **34**, 374–378 (2003).

63. S. Dorkenwald, C. E. McKellar, T. Macrina, N. Kemnitz, K. Lee, R. Lu, J. Wu, S. Popovych, E. Mitchell, B. Nehoran, Z. Jia, J. Alexander Bae, S. Mu, D. Ih, M. Castro, O. Ogedengbe, A. Halageri, K. Kuehner, A. R. Sterling, Z. Ashwood, J. Zung, D. Brittain, F. Collman, C. Schneider-Mizell, C. Jordan, W. Silversmith, C. Baker, D. Deutsch, L. Encarnacion-Rivera, S. Kumar, A. Burke, D. Bland, J. Gager, J. Hebditch, S. Koolman, M. Moore, S. Morejohn, B. Silverman, K. Willie, R. Willie, S. Yu, M. Murthy, H. Sebastian Seung, FlyWire: Online community for whole-brain connectomics. *Nat. Methods* **19**, 119–128 (2022).

64. S. Min, H. S. Chae, Y. H. Jang, S. Choi, S. Lee, Y. T. Jeong, W. D. Jones, S. J. Moon, Y.J. Kim, J. Chung, Identification of a peptidergic pathway critical to satiety responses in *Drosophila*. *Curr. Biol.* **26**, 814–820 (2016).

65. A. Hussain, H. K. Ucpunar, M. Zhang, L. F. Loschek, I. C. Grunwald Kadow, Neuropeptides modulate female chemosensory processing upon mating in *Drosophila*. *PLOS Biol.* **14**, e1002455 (2016).
